# Genetic and enzymatic basis of xylooligosaccharide metabolism by *Bifidobacterium longum*

**DOI:** 10.1080/19490976.2026.2701440

**Published:** 2026-07-10

**Authors:** Lisa Friess, Fionnuala M. McAuliffe, Paul D. Cotter, Jose Munoz-Munoz, Anthony L. Shiver, Kerwyn Casey Huang, Anne de Jong, Douwe van Sinderen

**Affiliations:** a APC Microbiome Ireland & School of Microbiology, University College Cork, Western Road, Cork, Ireland; b UCD Perinatal Research Centre, School of Medicine, University College Dublin, National Maternity Hospital, Dublin, Ireland; c Teagasc Food Research Centre, Cork, Ireland; d Department of Biosciences, Durham University, Durham, England, Great Britain; e Department of Bioengineering, Stanford University, Stanford, USA; f Department of Microbiology and Immunology, Stanford University School of Medicine, Stanford, USA; g Chan-Zuckerberg Biohub, San Francisco, USA; h Department of Molecular Genetics, University of Groningen, Groningen, the Netherlands

**Keywords:** Dietary fibre, XOS, probiotic, prebiotic, bifidobacteria, gut microbiota

## Abstract

Bifidobacteria are common members of the human gut microbiota and are associated with host health. *Bifidobacterium longum* subsp. *longum* (*B. longum*) is prevalent across host ages and can utilise diverse plant-derived glycans, including xylooligosaccharides (XOS), that are indigestible by humans. Here, we show that XOS utilisation is strain specific among members of *B. longum*. In *B. longum* NCIMB 8809, growth on XOS induced transcription of genes encoding three glycoside hydrolases (XouA, XouB, and XouC), together with adjacent genes (*xouDEF*) predicted to encode an ABC-type carbohydrate uptake system. Biochemical analyses demonstrated that XouA and XouC are β-xylosidases, whereas XouB is an α-arabinofuranosidase. Genetic disruption and complementation experiments showed that XouA and the XouDEF uptake system are required for growth on XOS. Together, these findings identify the genetic and enzymatic basis of XOS utilisation in *B. longum* and highlight how strain-level variation in carbohydrate metabolism may inform the design of targeted prebiotic and synbiotic strategies to promote gut health.

## Introduction

Xylooligosaccharides (XOS) are partially hydrolyzed fragments of xylans, a class of dietary fibers commonly found in vegetables and fruits that constitute part of a healthy human diet. XOS are composed of variable numbers of D-xylopyranosyl units connected by β-1,4 linkages.^1^ Depending on the xylan source, additional residues can be attached to the xylose backbone, as in glucuronoxylan, which is substituted with 1,2-linked 4-O-methyl–D-glucurono-pyranosyl groups (MeGlcA), and arabinoxylan, which is decorated with α-1,2- and/or α-1,3-linked L-arabinofuranose. XOS are commercially produced from xylan-rich lignocellulosic materials such as corn cobs, rice, or oats.^2^ Production typically involves physical (e.g., milling), chemical (NaOH treatment), and/or thermal pretreatment (hot-water extraction) to remove lignin and increase xylan accessibility,^3^ ^,^ ^4^ followed by enzymatic hydrolysis with endo-1,4-β-xylanases and purification of the resulting XOS. The final product generally contains a mixture of linear XOS with varying degrees of polymerization (DOP), most commonly between two and five, and up to seven.^5^

As with many other complex soluble dietary fibers, the human digestive system lacks the carbohydrate-active enzymes (CAZymes) required to digest xylan and XOS. These glycans therefore reach the colon largely intact,^6^ ^,^ ^7^ where they can be utilized by specific members of the resident microbiota, promoting to proliferation of responsive strains.^8^ ^,^ ^9^ Owing to their proposed prebiotic activity, XOS are increasingly used in the functional food industry.^10^ ^,^ ^11^ Clinical studies have shown that dietary XOS supplementation increases the relative abundance of the genus *Bifidobacterium.* ^8^ ^,^ ^9^ More broadly, the ability of probiotic or commensal strains to utilize dietary fibers is thought to be an important factor in microbiota modulation.^12^ For example, dietary resistant starch increases the relative abundance of *Bifidobacterium adolescentis,* ^13^ arabinoxylan supplementation specifically increases the relative abundance of *Bifidobacterium longum* subsp. *longum* (hereafter, *B. longum*),^14^ ^,^ ^15^ and arabino xylooligosaccharides increase the abundance of multiple bifidobacterial species.^16^ Such microbiota alterations have been associated with health benefits including improved immune function, antimicrobial activity, and enhanced bowel movement.^14^ ^,^ ^17-19^ *B. longum* strains, directly or indirectly, can promote short-chain fatty acid production, particularly acetate and butyrate, and strengthen the intestinal barrier.^20^ Clinical studies have also reported that *B. longum* supplementation can improve markers associated with type 2 diabetes and obesity, including triglycerides, fasting glucose, insulin, and glycated hemoglobin.^21^ ^,^ ^22^

*B. longum* is a human gut commensal capable of utilizing diverse plant-derived oligosaccharides.^7^ Like other bifidobacteria, its genome encodes a variety of CAZymes that enable the hydrolysis and metabolism of poly- and oligosaccharides. For substrates with a high degree of polymerization (DOP > 6,^23-25^) extracellular or cell wall-associated enzymes are generally required to reduce the DOP, allowing bifidobacteria to transport smaller oligosaccharides into the cell for intracellular degradation to monosaccharides. These monosaccharides are then channeled into the bifidobacteria-specific central energy-generating metabolic pathway known as the bifid shunt.^26^ Dietary polysaccharides degraded by extracellular bacterial enzymes can also be released into the gut environment and become available to both the enzyme-producing organism and other gut bacteria, a phenomenon known as cross-feeding. Cross-feeding is likely common in the gut and has been demonstrated for *Bacteroides ovatus* HM222, an extracellular enzyme producer, and *B. longum* PT4, a cross-feeder, where the combination allows *B. longum* to grow in media containing xylan as the sole carbon source.^27^ In this interaction, *B. ovatus* is thought to degrade xylan extracellularly to release XOS, initially with DOP > 5 and later smaller fragments (DOP 2-6), which can be used by secondary degraders such as *B. longum.* ^27-29^ A similar interaction has been described between *Bifidobacterium pseudocatenulatum* and *B. longum*, in which an extracellular endo-1,4-β-xylanase of the GH10 family produced by *B. pseudocatenulatum* releases XOS from long-chain xylan, enabling uptake and fermentation by *B. longum.* ^30^

Several enzymes with activity on XOS have been described, primarily in *B. adolescentis* (Table S1). Comparative studies of *B. longum* strains have identified apparent homologs of such enzymes; however, some strains harboring these homologs are unable to grow with XOS as the sole carbon source.^31^ Thus, it remains unclear how widespread XOS utilization is among *B. longum* strains and which enzymatic pathways support this phenotype. Here, we address this knowledge gap using transcriptomic, *in silico*, biochemical, and genetic analyzes to identify the enzymatic machinery required for XOS metabolism by *B. longum*.

## Materials and methods

### Strains, growth media, and cultivation conditions

Bacterial strains and plasmids utilized in this study are listed in Supplemental Table S2. *B. longum* strains were cultivated in modified de Man, Rogosa, and Sharpe medium prepared from individual components without a carbohydrate source (mMRS^32^) supplemented with 0.06% cysteine-HCl (w/v; Sigma-Aldrich, Germany) and, unless stated otherwise, 0.5% lactose (w/v; Sigma-Aldrich, Germany). Cultures were incubated anaerobically at 37 °C in a modular atmosphere-controlled system (Davidson and Hardy, UK). *Escherichia coli* strains were routinely cultivated in BD Difco™ lysogeny broth (LB; UK) at 37 °C with agitation at 180 rpm. Where appropriate, growth media were supplemented with kanamycin (Kan; 100 μg ml^−1^ for *E. coli*), erythromycin (Erm; 250 μg ml^−1^ for *E. coli* and 100 μg ml^−1^ for *B. longum*), or tetracycline (Tet; 10 μg ml^−1^ for *E. coli* and 5 μg ml^−1^ for *B. longum*).

### Growth measurements

Carbohydrate utilization by bifidobacterial strains was examined in mMRS medium supplemented with cysteine-HCl (0.06% w/v; Sigma-Aldrich, Germany) and the indicated carbohydrate (0.5%, w/v). The carbohydrates assessed were lactose, xylose (Fluka, Switzerland), xylan from beechwood (Megazyme, Ireland), xylitol (Sigma-Aldrich, Germany), and xylooligosaccharides (XOS). XOS preparations were kindly provided by Shandong Longlive Bio-Technology Co., China (referred to here as XOS-SL), or by van Wankum Ingredients and Henan Heagreen Bio-technology Co., Ltd., China (referred to here as XOS-HG). To quantify bacterial growth, 5 ml of freshly prepared mMRS medium supplemented with the indicated carbohydrate were inoculated with 50 μl of an overnight culture grown in mMRS supplemented with 0.06% cysteine-HCl (w/v) and 0.5% lactose (w/v), diluted to an OD_600 nm_ of ~1. Uninoculated mMRS medium and/or medium without supplemented carbohydrate were used as negative controls. Cultures were incubated anaerobically at 37 °C and OD_600 nm_ was measured after 24 h using a spectrophotometer. Assays were performed in triplicate.

### Gene-trait matching analysis

BLASTP- and BLASTN-mediated searches^33^ were performed against 25 complete genomes of *B. longum* strains previously isolated as part of the MicrobeMom study^34^ ^,^ ^35^ as well as two publicly available *B. longum* genomes, NCIMB 8809 and JCM 1217, to determine the presence or absence of specific genes. After growth measurements and genome analyzes were completed, genotype–phenotype gene-trait matching (GTM) was performed by correlating gene presence or absence with the ability of each strain to grow on XOS. This analysis was restricted to genes that exhibited increased transcription during growth on XOS.

### Transcriptome analysis

*B. longum* NCIMB 8809 was grown overnight in mMRS supplemented with 0.06% cysteine-HCl and 0.5% lactose, and then subcultivated with a 1% inoculum in mMRS supplemented with 0.06% cysteine-HCl and 0.5% lactose, xylan, or XOS. Cultures were grown to an OD_600 nm_ of 0.5–0.8, corresponding to mid-logarithmic phase. Four milliliters of each culture were centrifuged (Dlab D3024R) at 5500*g* for 4 min, and the harvested cells were stored at –80 °C. Biological duplicates were prepared for each condition. Cell pellets were shipped on dry ice to the Department of Molecular Genetics, University of Groningen, where RNA extraction, rRNA depletion, library construction, and RNA sequencing were performed. Ribosomal RNA was depleted using a RiboCop rRNA depletion kit (Lexogen, Austria), and libraries were prepared for Illumina sequencing using an NEBNext Ultra II Directional RNA Library Prep kit (New England Biolabs, UK).

Sequencing was performed on an Illumina NextSeq 1000, generating 100-bp single-end reads with an average depth of 8–12 million reads per sample. Read quality was assessed using FastQC v. 0.11.9 (Babraham Bioinformatics, Cambridge), and reads were mapped to the *B. longum* NCIMB 8809 reference genome (GCF_001446255.1) using Bowtie2 v. 2.4.2 with default settings. The resulting SAM files were converted to BAM format using SAMtools v. 1.11, and gene counts were obtained using featureCounts v. 2.0.1 (Subread/2.0.2). Differential expression analysis was performed using T-REx2.^36^

### Generation of gene disruption mutants

Targeted gene disruption was performed insertion of a non-replicating plasmid into the chromosome at a specific location by homologous recombination, thereby disrupting the coding region and predicted functionality of the targeted gene. To generate disruption mutants, internal fragments of *xouA*, *xouB*, *xouC*, or *xouF* were amplified by PCR using Q5 High-Fidelity DNA polymerase, and *B. longum* NCIMB 8809 chromosomal DNA as template. Primer sequences and fragment locations are listed in Table S4. PCR products were digested with HindIII and XbaI (New England Biolabs, UK), then ligated into pFREM2 digested with the same enzymes. The resulting ligation mixture was introduced into *E. coli* EC101 by chemical transformation.^37^ *E. coli* EC101 derivatives containing the expected recombinant pFREM2 constructs were selected on LB agar containing Erm. After verification by sequencing (Genewiz, Germany), recombinant plasmids pFREM2:*xouA*, pFREM2:*xouB*, pFREM2:*xouC*, and pFREM2:*xouF* were extracted using a GeneJET Plasmid Maxiprep kit (Fisher Scientific, US).

The recombinant plasmids were introduced into *B. longum* NCIMB 8809 via electroporation, as previously described,^38-40^ to achieve gene disruption by homologous recombination. Briefly, a single colony was grown in MRS broth (Difco, Fisher Scientific, US) at 37 °C for 16 h, then subcultured twice in MRS broth supplemented with 7% sucrose (Fisher Scientific, US) and 34 μg ml^−1^ iron(II) sulfate heptahydrate (Fisher Scientific, US) until an OD_600 nm_ of 0.9–1.1 was reached. This culture was then used to inoculate, at 10%, 50 mL of MRS broth reconstituted from individual components and supplemented with 1% lactose (Sigma-Aldrich, US), 7% sucrose, and 20 mM NaCl (Fisher Scientific, US). Cells were grown to an OD_600 nm_ of 0.35–0.5, harvested by centrifugation for 10 min at 4,500*g*, and washed three times with 2 mL of 0.5 M sucrose in 1 mM citrate buffer (pH 5.8). Wash steps were performed in 2 mL tubes by centrifugation for 1 min at 15,000*g*). Cells were then resuspended in 200 μl of 0.5 M sucrose in 1 mM citrate buffer. Transfer steps were carried out in an anaerobic chamber.

Competent cells and plasmid DNA were incubated on ice for 30 min in the anaerobic chamber before electroporation (2.5 V, 20 kW, 300 Ω). Cells were recovered in MRS broth modified with 100 ng ml^−1^ CaCl_2_ and 50 ng ml^−1^ 1,4-dihydroxy-2-naphthoic acid (DHNA^41^; Sigma-Aldrich, US) for 3 h at 39 °C. Transformants were selected on reinforced clostridial agar plates (RCA; Thermo Scientific, UK) supplemented with 100 µg ml^−1^ Ery, 100 ng ml^−1^ CaCl_2_, and 50 ng ml^−1^ DHNA after 48 h of growth at 39 °C. The genetic integrity of all gene disruption mutants (NCIMB 8809-Δ*xouA*, NCIMB 8809-Δ*xouB*, NCIMB 8809-Δ*xouC*, and NCIMB 8809-Δ*xouF*) was verified by Oxford Nanopore Technologies-mediated whole genome sequencing performed by Plasmidsaurus, US.

### Construction of complementation and expression vectors

For phenotypic complementation of the gene disruption mutants *B. longum* NCIMB 8809-Δ*xouA* and *B. longum* NCIMB 8809-Δ*xouF*, as well as the naturally occurring mutation in the *xouD* homolog present in *B. longum* strain MM0289, the *xouA, xouD*, and *xouF* genes were individually amplified by PCR using Q5 High-Fidelity DNA polymerase (New England Biolabs, UK). Native promoter regions were included where possible. Primer sequences, locus tag numbers, genomic locations, and associated promoters are listed in Table S5. PCR products were ligated into pBM5^38^ ^,^ ^42^ using ApaI and XhoI restriction sites (New England Biolabs, UK). The resulting plasmids were introduced into *E. coli* DH5α as a cloning host^37^ before transfer into EC101 cells harboring pNZEM. All constructs were verified by DNA sequencing (Plamidsaurus, US).

Plasmids were extracted from their *E. coli* host using a GeneJET Plasmid Miniprep kit (Thermo Scientific, UK) and introduced by electroporation, as described above, into *B. longum* NCIMB 8809, its isogenic disruption mutants, or *B. longum* MM0289. Transformants were selected on RCA plates (Thermo Scientific, UK) supplemented with 10 µg ml^−1^ Tet, 100 ng mL^−1^ CaCl_2_, and 50 ng mL^−1^ DHNA after 48 h of growth at 39 °C. Successful transformation was verified by colony PCR using primers targeting the multiple cloning site of the plasmid and the complementing gene (OneTaq 2X master mix; New England Biolabs, UK). Growth phenotypes of the complemented disruption mutants were assessed as described above.

### Assessment of cross-feeding interactions between *Bacteroides ovatus* and *B. longum* on xylan

Cross-feeding interactions between *Ba. ovatus* CCUG 4943 and *B. longum* NCIMB 8809 were assessed as follows. *Ba. ovatus* CCUG 4943 was grown overnight in Brain Heart Infusion medium (BHI; Oxoid, UK) supplemented with 0.06% cysteine-HCl (w/v), 5 μg ml^−1^ hemin (Sigma-Aldrich, Germany) and 1 μg ml^−1^ vitamin K1 (Sigma-Aldrich, Germany). *B. longum* strains were grown overnight in mMRS supplemented with 0.06% cysteine-HCl (w/v) and 0.5% lactose (w/v).

Freshly prepared modified reinforced clostridial medium (mRCM) lacking carbohydrate, consisting of 13 g l^−1^ yeast extract (Fisher Bioreagents, US), 10 g l^−1^ tryptone (Formedium, UK), 5 g l^−1^ NaCl (Fisher Bioreagents, US), and 3 g l^−1^ sodium acetate (Sigma-Aldrich, Germany), was supplemented with 0.5% lactose or xylan (w/v), 0.06% cysteine-HCl (w/v), 5 μg ml^−1^ hemin, and 1 μg ml^−1^ vitamin K1. Five milliliters of medium were inoculated with 50 μl of an overnight culture, diluted to an OD_600 nm_ of ~0.1, of either *Ba. ovatus* CCUG 4943, a *B. longum* strain, or a combination of both strains, to achieve an approximately 1:1000 dilution. Cultures were incubated anaerobically at 37 °C.

At 0, 4, 6, 8, and 24 h, samples were plated on selective media. *Ba. ovatus* CCUG 4943 was selected on BHI supplemented with 0.06% cysteine-HCl (w/v), 5 μg ml^−1^ hemin, 5 μg ml^−1^ vitamin K1, and 10 μg ml^−1^ vancomycin. *B. longum* NCIMB 8809 and its isogenic derivatives were selected on mMRS supplemented with 0.06% cysteine-HCl (w/v) 0.5% lactose (w/v), and 100 μg ml^−1^ mupirocin.

### Construction of protein overexpression vectors

For overexpression and purification of His-tagged proteins XouA, XouB, and XouC, the plasmids pET28b:XouA, pET28b:XouB, and pET28b:XouC were generated as follows. Details of recombinant plasmids, locus tags, genomic locations, and primers are listed in Table S3. DNA fragments encompassing the targeted genes were amplified with Q5 High Fidelity polymerase (New England Biolabs, UK) using chromosomal DNA, extracted with a Genelute bacterial genomic DNA kit (Sigma-Aldrich, Germany), as template. Amplicons were cloned into pET28b to incorporate an *N*-terminal His-tag (Novagen, now part of Merck KGaA, Germany), using NheI and EcoRI for *xouC* and NheI and NotI for *xouB* and *xouA*. All restriction enzymes were obtained from New England Biolabs, UK. *E. coli* BL21(DE3) cells were used as cloning hosts.^37^ All constructs were verified by DNA sequencing (Plasmidsaurus, US).

### Protein overproduction and purification

One hundred milliliters of NZY Auto-Induction LB medium (NZYtech, Portugal) were inoculated with 1% of the appropriate *E. coli* strain and incubated for 24 h at 24 °C with shaking at 300 rpm. Cells were harvested by centrifugation and resuspended in lysis buffer containing 50 mM Tris base (Fisher Bioreagents, US), 300 mM NaCl (Fisher Bioreagents, US), 50 mM CaCl_2_ (Fisher Bioreagents, US), and 10 mM imidazole (Sigma-Aldrich, Germany). Cells were lysed with a mini-beadbeater (BiospecProducts, US) for three 1-min cycles, alternating with 1-min incubations at 4 °C. Protein purification was performed using His-tag affinity gravity-flow chromatography with Ni-NTA matrices according to the manufacturer’s instructions (Qiagen, Germany).

To assess the molecular weights of the recombinant XouA_His_, XouB_His_, and XouC_His_, sodium dodecyl sulfate-polyacrylamide gel electrophoresis (SDS-PAGE) was performed as previously described^43^ using a Pierce™ Unstained Protein Molecular Weight Marker (14.4–116 kDa; Thermo Scientific, UK). Gels were stained with Coomassie Brilliant Blue. Elution fractions of interest were concentrated and dialyzed into 20 mM sodium phosphate buffer (pH 7) using Amicon® Ultra centrifugal filters with a 30 kDa molecular weight cut-off (Merck Millipore, Merck KGaA, Germany). Protein concentrations were determined using a Qubit® Fluorometer (Thermo Scientific, UK) and corresponding protein assay kit (Thermo Scientific, UK).

### Computational and comparative genomic analyzes

The Artemis genome browser^44^ was used to visualize genetic features in the annotated genome of *B. longum* NCIMB 8809.^45^

Pangenome analysis and core-genome alignment were performed using Roary v. 3.13.0^46^ with the -e, -*n*, and –mafft flags, using a BLASTP identity threshold of 90% and defining core genes as those present in ≥99% of genomes. The resulting core-genome alignment was used to generate a maximum-likelihood phylogeny using IQtree v. 2.2^47^ with automatic model selection by ModelFinder. Branch support was assessed using 1,000 ultrafast bootstrap replicates. The phylogenetic tree was visualized using iTOL.^48^

Protein function and homology searches were performed using BLASTP,^33^ HHpred,^49^ and HMMER.^50^ DeepLoc-2.1^51^ was used to predict whether protein sequences were secreted or cytoplasmic. The CAZy database^52^ was used to determine GH43 subfamily classifications. AlphaFold3 was used to predict the structures of relevant proteins described in this study.^53^ Default server parameters were used, with five recycling cycles to refine each model. Predicted template modeling (pTM) scores were above 0.5 in all cases, indicating that the computed models were suitable for structural interpretation.

For gene cluster alignments, genome sequences were obtained from NCBI on 25 September 2025, and regions were identified using tBLASTn analysis with cut-off criteria of at least 60% amino acid identify across at least 75% coverage and an E-value < 10^−20^. Cluster alignments were generated using the Comparative Gene Cluster Analysis Toolbox, Clinker-Cagecat.^54^ ^,^ ^55^ To analyze additional human-associated bifidobacterial species, tBLASTn analysis was performed against a database of 489 bifidobacterial genomes representing 11 species or subspecies previously isolated and sequenced from an Irish mother-infant cohort as part of the MicrobeMom study.^34^ ^,^ ^35^

### Enzymatic activity assays

Purified enzymes were assayed using the following synthetic pNP substrates (Carbosynth, UK): pNP-α-L-arabinofuranose, pNP-α-L-arabinopyranose, and pNP-β-D-xylopyranose. Reactions were carried out at 37 °C in 20 mM potassium phosphate buffer (pH 7) containing 2, 4, 5, 7.5, or 10 mM substrate and 0.1 µM purified enzyme. Enzymatic activity was quantified by measuring absorbance at 405 nm every 30 s for 5 min using a spectrophotometer.

Enzymatic reactions using synthetic XOS and arabinoxylo-oligosaccharides were performed in 20 mM potassium phosphate buffer (pH 7) at 37 °C. The following substrates were used (Megazyme, Ireland): xylobiose, xylotriose, xylotetraose, and xylopentaose, each containing β-1,4 linkages; 3^2^-α-L-arabinofuranosyl-xylobiose (A3X; α-1,3 and β-1,4 linkages); 2^3^-α-L-arabinofuranosyl-xylotriose (A2XX; α-1,2 and β-1,4 linkages); 2^3^-3^3^-di-a-L-arabinofuranosyl-xylotriose (A23XX; α-1,2, α-1,3 and β-1,4 linkages); and 3^2^-a-L-arabinofuranosyl-arabinotriose (AA3A; α-1,3 and α-1,5 linkages). Substrates were tested at 0.5, 1, and 2 mg ml^−1^. Enzyme concentrations were 0.5 µM for XouC_His_ and XouB_His_ and 0.1 µM for XouA_His_. Released monomeric D-xylose was measured using a D-xylose assay kit (Megazyme, Ireland), and released L-arabinose was measured using an L-arabinose/D-galactose assay kit (Megazyme, Ireland). Absorbance was measured every 30 s for 5 min. Enzymatic reactions were performed in duplicate.

*K*_cat_/*K*_m_ values were determined from the slope of the linear regression of initial velocity against substrate concentration, normalized by enzyme concentration.

### HPAEC-PAD carbohydrate analysis

HPAEC-PAD analysis was performed using a Dionex ICS-3000 system (Thermo Scientific, US). Carbohydrate fractions from the hydrolysis assays described above were analyzed as 200 μl aliquots. Samples were separated on a CarboPac PA1 analytical anion-exchange column (250 mm × 4 mm) equipped with a CarboPac PA1 guard column (50 mm × 4 mm) and visualized using a pulsed electrochemical detector (ED40) in PAD mode (Thermo Scientific Dionex, US).

Elution was performed with 100 mM potassium hydroxide at a constant flow rate of 0.063 ml min^−1^ at 30 °C. A 10-min run was used for analysis of enzymatic assays and cell-free supernatants, whereas a 120-min run was used for XOS compositional analysis. Chromatographic profiles of the tested carbohydrates and, where available, their putative breakdown products were used as references for quantitative analysis of glycan breakdown by XouA_His_ and XouB_His_. These profiles were also used for analysis of cell-free supernatants from growth medium collected after 24 h of growth on XOS by *B. longum* NCIMB 8809 and the gene disruption mutants NCIMB 8809-Δ*xouA* and NCIMB 8809-Δ*xouC*. Chromeleon v. 7.3 (Dionex Corporation) was used to integrate and evaluate the chromatograms.

## Results

### XOS utilization varies among *B. longum* strains

**Figure 1.**
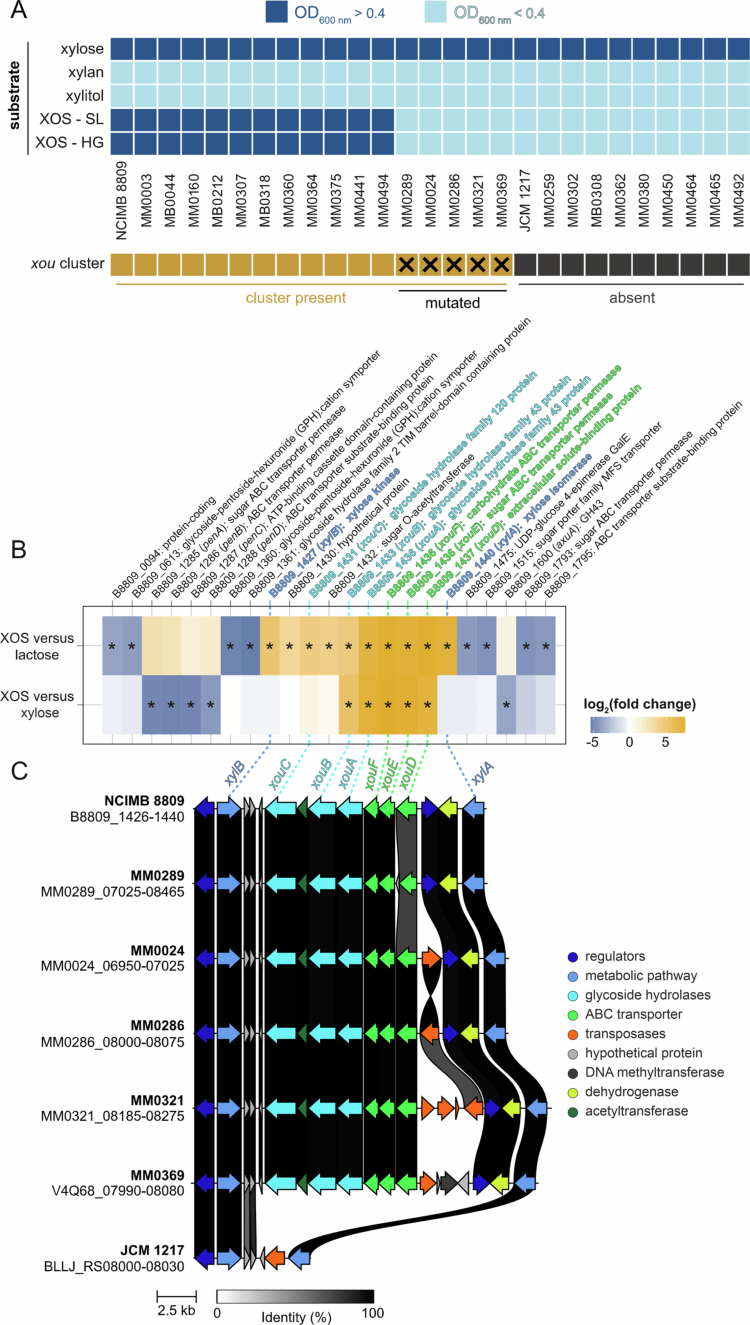
Growth and transcriptomic profiling identify the xyl-xou cluster involved in XOS metabolism in B. longum. (A) Top: growth of 27 *B. longum* strains after 24 h in mMRS medium containing xylose, xylan, xylitol, or one of two commercial XOS preparations as the sole carbohydrate sources. All strains grew on xylose to OD_600 nm_ > 0.4 (dark blue), whereas their OD_600 nm_ remained < 0.4 on xylan and xylitol (light blue). Twelve strains grew on both commercial XOS preparations. Bottom: all 12 XOS-utilizing strains contained the *xyl-xou* cluster in their genomes (orange), suggesting a link to XOS metabolism. Five additional strains also contained the *xyl-xou* cluster but carried mutations predicted to disrupt function (black crosses). (B) *B. longum* NCIMB 8809 genes differentially expressed during growth on XOS compared with lactose or xylose. Asterisks denote absolute log_2_(fold change) > 3.5 and –log_2_(*p*) > 5. (C) Genetic organization of the *xyl-xou* cluster across strains. NCIMB 8809 represents the 12 strains containing a complete *xyl-xou* cluster, and JCM 1217 represents the 10 strains lacking the *xou* cluster but retaining *xylA* and *xylB*. Colors indicate predicted gene function: glycoside hydrolase (light blue), ABC transporter (green), xylose metabolism (blue), transcriptional regulator (dark blue), transposase-encoding gene (orange), dehydrogenase (yellow), acetyltransferase (dark green), and DNA methyltransferase (dark gray).

We first investigated the ability of twenty-five human-derived *B. longum* strains^34^ ^,^ ^35^ and two publicly available *B. longum* strains to grow in mMRS medium containing xylose, xylitol, or xylose-containing plant-derived glycans as the sole carbohydrate source (Figure 1A). No appreciable growth was observed in mMRS medium without added carbohydrate (OD_600 nm_ < 0.1). All strains utilized xylose as the sole carbon source, whereas none exhibited substantial growth on xylan or xylitol. In contrast, only twelve of the 27 strains, including *B. longum* NCIMB 8809, exhibited substantial growth on XOS from either of two commercial sources (Methods); the remaining 15 strains were unable to grow on either XOS substrate. Thus, XOS utilization varies substantially among *B. longum* strains. All XOS-utilizing strains grew on both commercial preparations, likely reflecting their similar compositions: xylotriose and xylobiose were the most abundant components (21–24% each), followed by xylotetraose (7–10%), whereas xylopentaose and xylose were minor components (<5%; Fig. S1). Together, these results indicate that XOS utilization is a strain-specific phenotype in *B. longum*, motivating a search for the genetic determinants that distinguish XOS-utilizing from non-utilizing strains.

### Growth on XOS induces a candidate uptake and metabolism gene cluster

To identify genes involved in XOS metabolism, we focused on *B. longum* strain NCIMB 8809 and performed transcriptomic analysis during logarithmic growth in mMRS medium containing XOS as the sole carbohydrate source. We compared this condition with logarithmic growth in either lactose or xylose to distinguish genes specifically associated with XOS utilization from genes involved more generally in xylose metabolism (Figure 1B, S2A, B).

Genes significantly upregulated during growth on XOS compared with lactose were concentrated in a single gene cluster (locus tags B8809_1426–B8809_1440; Figure 1C), which we designate here as the *xyl-xou* cluster. This cluster includes genes encoding xylose kinase (*xylA*, B8809_1427) and xylose isomerase (*xylB*, B8809_1440), which we recently showed are required for xylose metabolism by converting xylose into xylulose-5-phosphate for entry into the bifid shunt.^26^ ^,^ ^38^ ^,^ ^56^ The *xyl-xou* cluster also contains three genes predicted to encode glycoside hydrolases (GHs): a GH120 enzyme encoded by *xouC* (B8809_1431) and two GH43 enzymes encoded by *xouB* (B8809_1433) and *xouA* (B8809_1434). Transcription of *xouC* was significantly upregulated only relative to lactose, whereas *xouB* and *xouA* were significantly upregulated relative to both lactose and xylose. The cluster also contains three adjacent genes, *xouDEF* (B8809_1435–B8809_1437), predicted to encode components of an ABC-type carbohydrate uptake system. Because *xouDEF* transcription was significantly higher during growth on XOS than during growth on either lactose or xylose, these genes are candidates for XOS-specific internalization.

Transcription of four adjacent genes, B8809_1285–B8809_1288, was significantly lower during growth on XOS than during growth on xylose, but slightly, although not significantly, higher during growth on XOS than during growth on lactose. These genes, previously named *penABCD*, are thought to encode an ABC transporter system responsible for the uptake of pentose sugars, including xylose.^56^ Three genes predicted to encode MFS transporters, B8809_0613, B8809_1360, and B8809_1515, were significantly downregulated during growth on XOS compared with lactose but were unaffected compared with xylose. These patterns are consistent with the prediction that B8809_1360 is involved in lactose and galactose uptake (74.7% identity to *lacS* in *B. breve*) and that B8809_1515 is involved in glucose uptake (98.7% identity to *glcP in B. longum* NCC2705).^57^

Together, these transcriptomic data identify the *xyl-xou* cluster as a candidate locus for XOS uptake and metabolism in *B. longum*, providing the basis for subsequent genetic and biochemical analyzes.

### Gene trait matching links XOS utilization to an intact *xyl-xou* cluster

We compared the presence or absence of NCIMB 8809 genes transcriptionally upregulated during growth on XOS or xylose across the other 26 *B. longum* strains for which XOS growth phenotypes were measured. Of the 27 strains, 10, including *B. longum* JCM1217, contained only a partial *xyl-xou* cluster lacking *xouA*, *xouB*, *xouC*, and *xouDEF*, and all were unable to utilize XOS (Figure 1A). The remaining 17 genomes contain clear homologs of these genes; however, only 12 of these strains were able to utilize XOS (Figure 1A). Compared to *B. longum* NCIMB 8809, the *xyl-xou* clusters from the five non-utilizing strains contain either transposon insertions or a point mutation (Figure 1A,C). Four of these strains, MM0024, MM0286, MM0321, and MM0369, contain one to four insertion elements between their *xouR* homolog and the *xouDEF* genes (Figure 1C). The fifth strain, MM0289, carries a single-nucleotide variant in the homolog of *xouD*, which encodes the predicted substrate-binding protein of the ABC transporter. This variant produces a nonsense mutation that introduces a premature stop codon at codon 364 of 438. These findings suggest that an intact *xyl-xou* cluster is required for growth on XOS.

A core-genome phylogeny of the 27 strains revealed that strains clustered independently of the presence or absence of the *xyl-xou* gene cluster (Fig. S3). This pattern suggests that the *xyl-xou* cluster was mutated and/or lost multiple times and that its presence is not restricted to a single strain lineage. Together, these genotype–phenotype associations point to the *xyl-xou* cluster as the likely determinant of strain-specific XOS utilization, motivating functional analysis of the encoded glycoside hydrolases and transporter components.

### Biochemical analyzes define enzymatic activities required for XOS metabolism

To gain further insight into the three GHs encoded by the *xyl-xou* cluster, we used HHPred, HMMER, and the CAZy database^49^ ^,^ ^50^ ^,^ ^52^ to analyze their predicted domains. XouA is predicted to contain two domains: an *N*-terminal GH43_11 catalytic domain with predicted β-xylosidase activity and a C-terminal CBM91 carbohydrate binding domain. XouB is predicted to contain a single GH43_12 domain, a family associated with α-arabinofuranosidase or β-xylosidase activity in the CAZy database. HHpred^49^ comparison with previously characterized enzymes further supported predicted α-arabinofuranosidase activity for XouB. XouC is a member of the GH120 family, with predicted β-xylosidase activity in CAZy. DeepLoc-2.1^51^ predicted that all three enzymes are intracellular.

**Figure 2.**
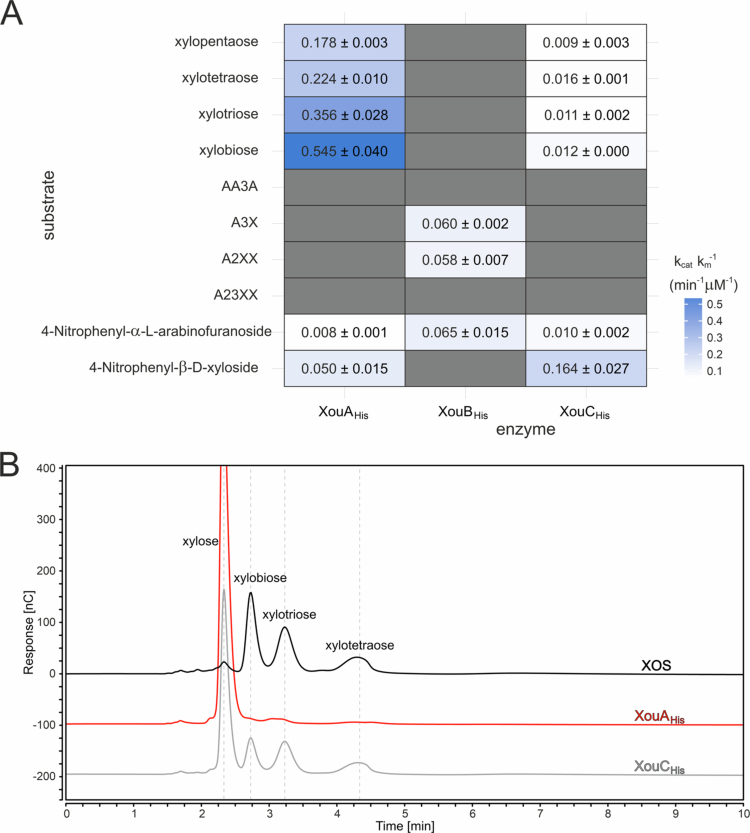
XouA and XouC exhibit β-xylosidase activity, while XouB acts as an α-arabinofuranosidase. (A)*K*_cat_/*K*_m_ values for XouA_His_, XouB_His_, and XouC_His_ incubated with pNP substrates, XOS, or AXOS. Enzymatic activity was quantified by measuring released pNP, xylose, or arabinose, respectively, over 5 min. (B) HPAEC-PAD analysis of XOS hydrolysis products after incubation of XOS alone (black) or XOS with XouA_His_ (red) or XouC (gray) for 5 min. Dashed lines indicate retention times of xylose, xylobiose, xylotriose, and xylotetraose standards.

To experimentally determine the activities of these three GHs, we individually cloned the corresponding genes into the pET28b expression vector for protein overexpression and purification. The apparent molecular weights of the purified His-tagged proteins, XouA_His_, XouB_His_ and XouC_His_, were consistent with their calculated molecular weights (Fig. S4). All three purified enzymes exhibited hydrolytic activity toward 4-nitrophenyl-L-arabinofuranose (Figure 2A), although their *K*_cat_/*K*_m_ values were lower than that of a previous characterized α-arabinofuranosidase.^38^ In addition, XouC_His_ and XouA_His_ exhibited hydrolytic activity toward 4-nitrophenyl-β-D-xylopyranose.

We next assessed enzyme activity on XOS and arabinoxylan-oligosaccharide substrates with varying linkages and DOP. XouC_His_ and XouA_His_ exhibited β-xylosidase activity on unbranched XOS, consistent with *in silico* predictions. XouA_His_ exhibited a significantly higher *K*_cat_/*K*_m_ than XouC_His_; however, neither enzyme released xylose from XOS substrates containing an arabinose side chain. XouB_His_ exhibited α-arabinofuranosidase activity on singly branched arabinoxylo-oligosaccharides containing either an α-1,2 or α-1,3 substitution, consistent with the HHPred prediction. However, no measurable release of monomeric arabinose was detected from the arabino-oligosaccharide containing an α-1,2 substitution. To further explore substrate activity, we tested XouC_His_ and XouA_His_ using commercially available XOS. At 1 µM enzyme concentration, XouA_His_ fully degraded 10 mg ml^−1^ XOS into xylose within 5 min, whereas 1 µM XouC_His_ required a substantially longer incubation time to fully degrade XOS (Figure 2B).

To investigate the structural basis of substrate specificity, we generated AlphaFold3 models of XouA, XouB, and XouC bound to their predicted ligands.^53^ The XouA model revealed an open active-site pocket that may accommodate substrates with larger DOP (Fig. S5A), consistent with the higher *K*_cat_/*K*_m_ value of XouA compared with the other two enzymes. Models of XouB and XouC bound to their ligands are shown in Fig. S5B and S5C, respectively. In XouB, a small loop near the arabinose moiety, comprising residues A218, V219, W220, and A221, may restrict the binding of longer residues while leaving O1 available for linkage to additional residues. In XouC, a longer and bulkier loop, comprising residues A499, D500, A501, A502, and G503, may contribute to the lower activity of XouC on the substrates tested (Figure 2A). Predicted catalytic residues were conserved in all three enzymes: D127 and E187 in XouA, D162 and E223 in XouB, and D387 and E411 in XouC (Fig. S5).

Together, these biochemical and structural analyzes indicate that the *xyl-xou* cluster encodes complementary intracellular enzymatic activities for XOS debranching and degradation, with XouA acting as the primary β-xylosidase on unbranched XOS and XouB providing α-arabinofuranosidase activity on branched substrates.

### Genetic analysis demonstrates that XouA and XouDEF are required for XOS utilization

**Figure 3.**
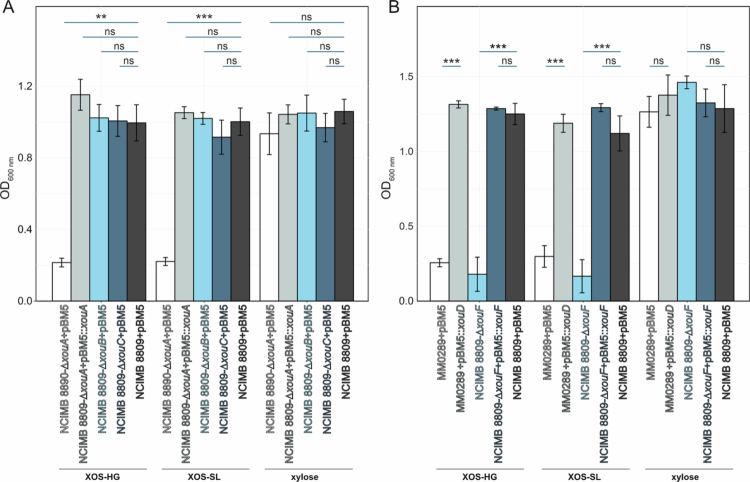
Genetic disruption of *xouA* or *xouF* prevents growth on XOS. OD_600 nm_ after 24 h of anaerobic growth in mMRS supplemented with 0.5% (w/v) XOS-HG, XOS-SL, or xylose. (A) Disruption of *xouA*, which encodes a glycoside hydrolase, prevents growth on XOS-HG and XOS-SL but not xylose. Complementation with pBM5:*xouA* restores growth on both XOS substrates. Disruption of *xouB* or *xouC* does not significantly affect growth on XOS or xylose. (B) Disruption of *xouF*, which encodes an ABC-type carbohydrate permease, prevents growth on XOS-HG and XOS-SL but not xylose. Complementation with pBM5:*xouF* restores growth on both XOS substrates. Introduction of pBM5:*xouD* restores growth of *B. longum* MM0289 on XOS. Asterisks indicate significant differences (*: *p* ≤ 0.,05, **: *p* ≤ 0.,005, ***: *p* ≤ 0.0005); ns indicates no significant difference.

We next generated gene disruption mutants to assess the importance of individual genes in the *xyl-xou* cluster in XOS utilization by *B. longum* NCIMB 8809. Disruption of *xouC* or *xouB* did not significantly affect growth on either of the two commercial XOS substrates (Figure 3A). In contrast, disruption of *xouA* significantly impaired growth on both XOS substrates after 24 h. Reintroduction of *xouA* on plasmid pBM5:*xouA* into NCIMB 8809-Δ*xouA* restored growth on XOS (Figure 3A).

To determine which XOS fractions remained after growth, aliquots of spent supernatant from wild-type NCIMB 8809, NCIMB 8809-Δ*xouC*, and NCIMB 8809-Δ*xouA* were analyzed by HPAEC-PAD after 24 h of growth on XOS. The carbohydrate profile of NCIMB 8809-Δ*xouA* was virtually identical to that of uninoculated XOS-containing growth medium, confirming loss of the ability to utilize XOS with DOP 2–5 (Fig. S6A), with future studies being required to determine the exact upper size limit of XOS uptake by XouDEF. Consistent with the observation that *xouC* disruption did not affect growth on XOS, the carbohydrate utilization profile of NCIMB 8809-Δ*xouC* was comparable to that of wild type, with xylotriose, xylotetraose, and possibly xylobiose (whose peak is partially obscured by a medium-derived peak) completely depleted after 24 h of growth (Fig. S6A). Thus, although XouC_His_ exhibits *in-vitro* activity on β-1,4 linked XOS, XouA appears to be the primary enzyme required for XOS utilization under these growth conditions.

Mutants in *xylAB* and in *penD*, which encodes a component of the xylose uptake system, were also tested for their ability to grow with XOS as the sole carbohydrate source.^56^ As expected, disruption of either *xylA* or *xylB* prevented XOS utilization, consistent with XOS degradation into xylose followed by XylA- and XylB-dependent conversion into intermediates that enter the bifid shunt^56^ (Fig. S6B). In contrast, *B. longum* NCIMB 8809-Δ*penD* grew with XOS as the sole carbohydrate source, indicating that *B. longum* NCIMB 8809 uses distinct ABC-type uptake systems for xylose and XOS internalization (Fig. S6B).

Because *xouF* is predicted to encode an ABC-type carbohydrate permease, we reasoned that disruption of *xouF* might prevent XOS uptake. Indeed, NCIMB 8809-Δ*xouF* + pBM5 was unable to grow with XOS as the sole carbon source. Complementation via pBM5:*xouF* restored growth on XOS to levels similar to those of wild type carrying the empty pBM5 plasmid after 24 h, validating the importance of *xouF* in XOS utilization (Figure 3B). This finding is consistent with the lack of XOS utilization by *B. longum* MM0289, which harbors a mutation in the *xouD* homolog predicted to encode an extracellular solute-binding protein. Introduction of an intact copy of *xouD* on plasmid pBM5:*xouD* into *B. longum* MM0289 reversed this strain’s inability to grow on XOS as the sole carbohydrate substrate (Figure 3B).

Together, these genetic analyzes demonstrate that XouA and the XouDEF ABC-type uptake system are required for XOS utilization by *B. longum* NCIMB 8809, explaining how disruption of the *xyl-xou* cluster can generate strain-level variation in XOS metabolism.

### XouA enables *B. longum* to cross-feed on xylan-derived oligosaccharides

**Figure 4.**
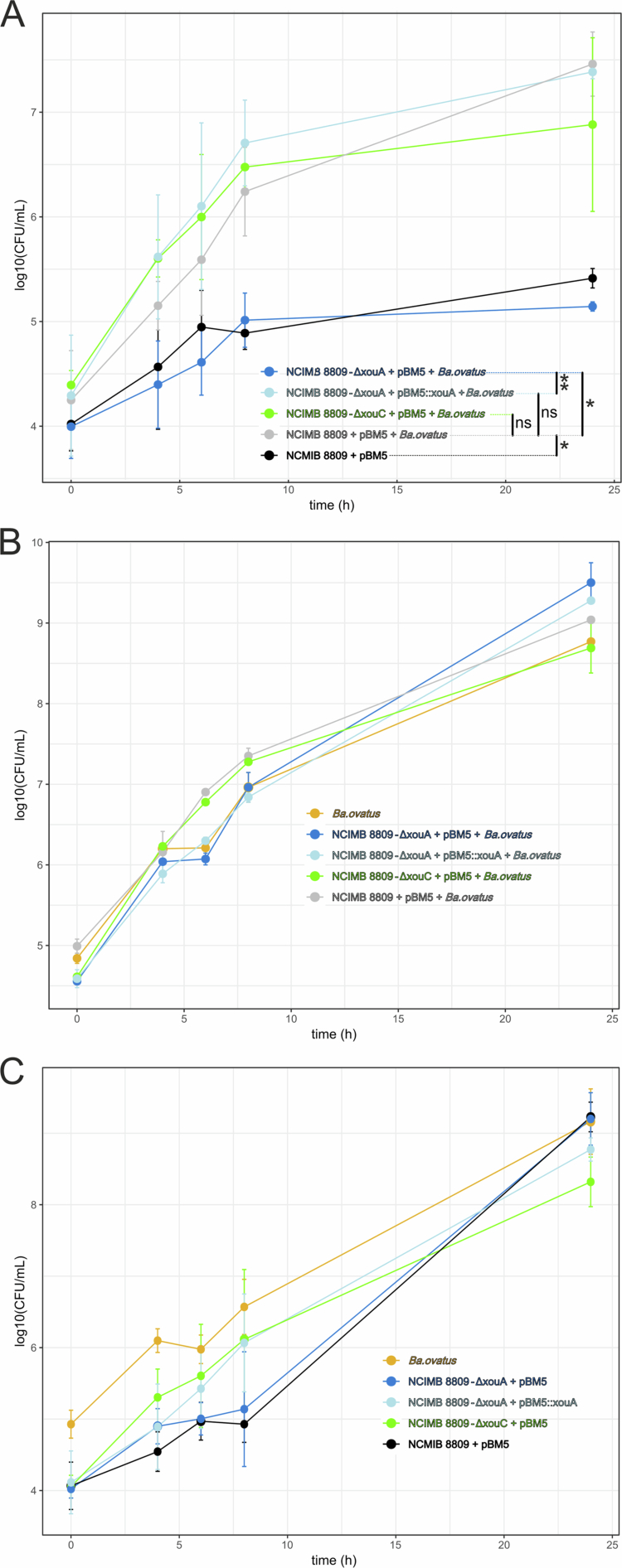
XouA is required for *B. longum* cross-feeding on *B. ovatus*-derived xylan oligosaccharides. (A) Growth of *B. longum* strains in co-culture with *Ba. ovatus* CCUG 4943 in mRCM supplemented with 0.5% xylan (w/v), 0.06% cysteine-HCl (w/v), 5 μg ml^−1^ hemin, and 1 μg ml^−1^ vitamin K1. Samples were plated on mMRS agar supplemented with 0.5% lactose (w/v), 0.06% cysteine-HCl (w/v), and 100 µgml^−1^ mupirocin to select for *B. longum*. (B) Growth of *B. ovatus* CCUG 4943 in the same co-cultures, quantified by plating on BHI agar supplemented with 0.06% cysteine-HCl (w/v), 5 μgml^−1^ hemin, 1 μgml^−1^ vitamin K1, and 10 μgml^−1^ vancomycin.(C) Growth of *B. longum* strains and *B. ovatus* CCUG 4943 as single cultures in mRCM supplemented with 0.5% lactose (w/v), 0.06% cysteine-HCl (w/v), 5 μg ml^−1^ hemin, and 1 μg ml^−1^ vitamin K1. Cultures were plated on the corresponding selective agar described above. Data is shown as log_10_(CFU ml^−1^) over time. Asterisks indicate significant differences; ns indicates no significant difference.

Cross-feeding between *Ba. ovatus* HM222 and *B. longum* PT4 during growth on xylan has previously been demonstrated.^27^ To determine whether the *xyl-xou* cluster is required for xylan-related cross-feeding between *B. longum* and a primary xylan degrader, we cultivated *B. longum* NCMIB 8809 together with *Ba. ovatus* CCUG 4943 in medium containing xylan as the sole carbohydrate source. The xylan degrading activity of *Ba. ovatus* CCUG 4943 enabled *B. longum* NCIMB 8809 carrying the empty’ pBM5 plasmid to grow in xylan-containing medium, presumably through cross-feeding (Figure 4A). In contrast, *B. longum* NCIMB 8809-Δ*xouA* carrying the empty pBM5 plasmid was unable to grow under the same co-cultivation conditions. Growth was restored by complementation with pBM5:*xouA*, and *B. longum* NCIMB 8809-Δ*xouC* carrying the empty pBM5 plasmid grew tolevels comparable to those of NCIMB 8809 carrying empty pBM5 (Figure 4A). Across co-cultures, *Ba. ovatus* CCUG 4943 grew at similar rates (Figure 4B), indicating that differences in *B. longum* growth were not attributable to altered growth of the primary xylan degrader. As a control, all tested *B. longum* strains were able to grow on lactose (Figure 4C).

Together, these findings demonstrate that xylan-mediated cross-feeding by *B. longum* NCIMB 8809 in the presence of *Ba. ovatus* CCUG 4943 depends on *xouA* and, therefore, on the ability of *B. longum* to metabolize XOS released from xylan.

### The *xyl-xou* gene cluster is conserved across multiple human-associated *Bifidobacterium*species

**Figure 5.**
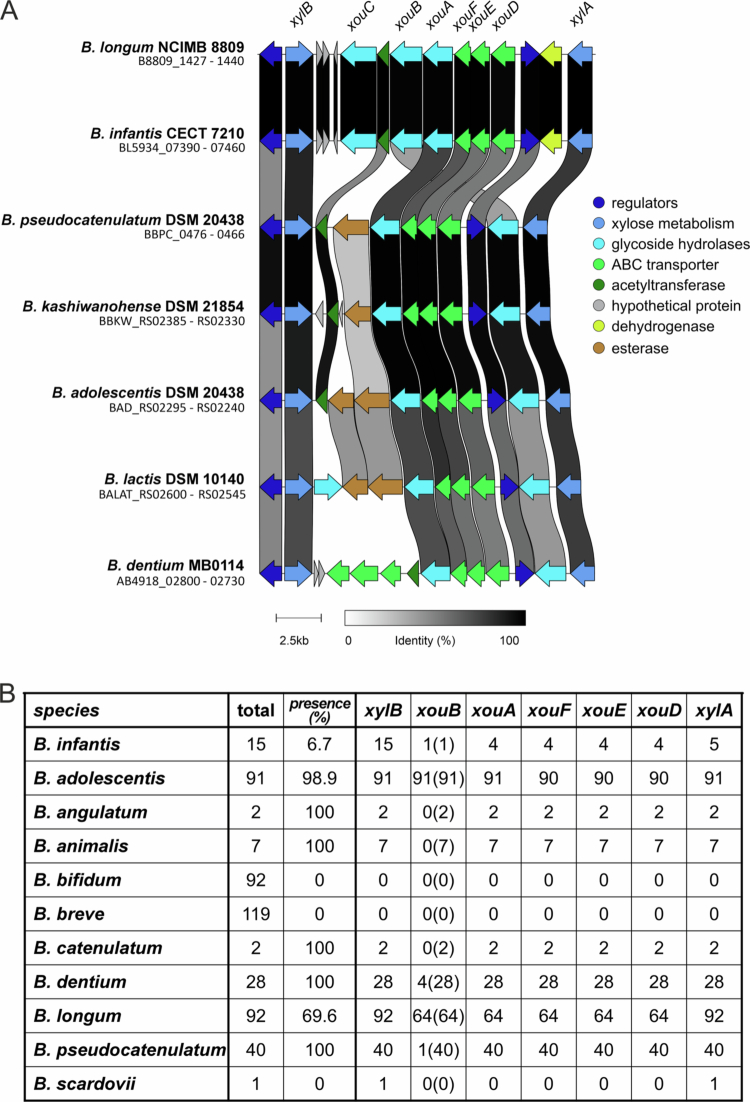
Conservation and diversification of the *xyl-xou* cluster across bifidobacterial species. (A) Genetic organization of *xyl-xou* clusters across representative bifidobacterial genomes. Colors indicate gene function: glycoside hydrolase (light blue), xylose metabolism (blue), transcriptional regulator (dark blue), ABC transporter (light green), acetyltransferase (dark green), dehydrogenase (yellow), and esterase (brown). Genome sequences were obtained from NCBI on 25 September 2025. (B) Distribution of homologs corresponding to conserved genes in the *B. longum* NCIMB 8809 *xyl-xou* gene cluster across bifidobacterial strains isolated as part of the MicrobeMom study,^34^ ^,^ ^35^ identified using BLAST analysis (Methods). The “presence (%)” column indicates the percentage of strains within each species containing homologs of the *xyl-xou* cluster. Numbers in parentheses for *xouB* indicate homologs corresponding to *B. adolescentis* DSM 20083.

To assess whether other human-associated bifidobacterial species contain homologs of the *xyl-xou* gene cluster, we performed a tBLASTn analysis against a database of 489 genomes representing 11 species or subspecies.^34^ ^,^ ^35^ Regions were identified using cut-off criteria of at least 60% amino acid identify across at least 75% coverage and an E-value < 10^−20^. Six publicly available genomes were included for cluster alignment (Figure 5A).

We found partial or complete homologs of the *xyl-xou* cluster in six additional bifidobacterial species: *B. longum* subsp. *infantis*, *B. adolescentis*, *B. angulatum*, *B. animalis* subsp. *lactis*, *B. catenulatum*, *B. dentium*, and *B. pseudocatenulatum*. In these genomes, the homologous cluster encodes two GH43 enzymes and an ABC-type transporter system and is flanked by the xylose metabolic genes *xylA* and *xylB*. Although the clusters in *B. angulatum*, *B. catenulatum*, *B. dentium*, and *B. pseudocatenulatum* harbor a *xouB* homolog, its similarity to *B. longum xouB* sequence was below the cut-off values used for the broader search.

Homologs of *xouC*, which encodes a GH120 enzyme, appear to be uniquely present in certain *B. longum* strains. In contrast, the *xyl-xou* cluster homologs of *B. catenulatum* and *B. pseudocatenulatum* each harbor one predicted esterase gene, whereas those of *B. adolescentis* and *B. animalis* subsp. *lactis* each harbor two predicted esterase genes; such genes are absent from the *B. longum xyl-xou* cluster. Partial loss and variable presence of the *xyl-xou* cluster appear to be specific to *B. longum*: with the exception of the single *B. scardovii* isolate and one *B. adolescentis* strain, the *xyl-xou* cluster, when present in a species, was conserved across all assessed strains of that species (Figure 5B).Together, these comparative genomic analyzes indicate that the *xyl-xou* cluster belongs to a broader family of bifidobacterial XOS utilization loci, but that *B. longum* exhibits unusual strain-level variation in cluster integrity and presence.

## Discussion

The diversity of CAZymes encoded by *Bifidobacterium* species and strains likely contributes to their abundance and persistence across host life stages. For example, *Bifidobacterium bifidum* is adapted to utilize human milk oligosaccharides (HMOs), supporting its abundance and prevalence in breast-fed infants, whereas *Bifidobacterium adolescentis* is associated with the metabolism of plant-based carbohydrates common in the adult diet.^58^ ^,^ ^59^ *B. longum* is among the most prevalent bifidobacterial species across host ages: *B. longum* subsp. *infantis* and *B. longum* subsp. *iuvenis* are avid HMO utilisers and are consequently enriched in the infant gut,^60^ whereas *B. longum* subsp. *longum*, which is detected in fecal samples from both infants and adults, appears to specialize in the metabolism of plant-derived glycans, including XOS.^61^ However, the genetic and enzymatic basis of XOS utilization has remained unclear.

Here, we identify a *xyl-xou* locus that mediates XOS uptake and metabolism in *B. longum*. This cluster is variably present among *B. longum* strains and has homologs in several human-associated bifidobacterial species, including *B. adolescentis* and *B. pseudocatenulatum*. Genetic analysis demonstrated that an ABC-type carbohydrate uptake system encoded by *xouDEF* is required for XOS utilization, while biochemical and genetic analyzes identified XouA as the primary β-xylosidase required for degradation of short-chain, unbranched XOS. Together, these findings define a strain-specific pathway for XOS metabolism in *B. longum* and provide a mechanistic explanation for why some strains can grow on XOS whereas others cannot.

Dietary fibers strongly influence gut microbiota composition, and xylan-derived oligosaccharides have repeatedly been associated with increased relative abundance of bifidobacteria.^8^ However, xylan is structurally complex. Depending on its plant source and extraction method, xylan can contain glucuronic acid, arabinose, xylose, and/or galactose substitutions, generating oligosaccharides that differ in size, branching, and linkage structure. These biochemical differences likely determine which bacterial enzymes and transport systems are required for utilization. Consistent with this idea, none of the *B. longum* strains tested here grew on polymeric xylan, whereas several, but not all, grew on shorter XOS. Thus, *B. longum* appears to rely on other gut microbes to first depolymerize xylan extracellularly, after which strain-specific uptake and intracellular metabolism determine whether a given *B. longum* strain can benefit from the released oligosaccharides.

The variable ability of *B. longum* strains to utilize XOS highlights substantial metabolic heterogeneity within this species. Unlike a model in which all *B. longum* strains share the same dietary niche, our findings suggest that individual strains differ in their capacity to access specific plant-derived carbohydrates. This variation may reflect adaptation to ecological contexts in which particular glycans are more or less available, including differences in diet, geography, seasonality, or the presence of primary degraders that release XOS from xylan. Notably, XOS utilization did not appear to depend simply on whether a strain was isolated from an infant or an adult. Infant-associated *B. longum* strains carrying an intact *xyl-xou* cluster may therefore be better positioned to respond to dietary transitions during weaning, potentially contributing to the broad prevalence of *B. longum* across host ages.

Our biochemical analyzes indicate that XouA and XouC are β-xylosidases, while XouB is an α-arabinofuranosidase. XouA showed stronger activity on unbranched XOS than XouC and was required for growth on XOS with low DOP, indicating that XouA is the primary β-xylosidase under the conditions tested here. XouC elicited β-xylosidase activity *in vitro* but was dispensable for growth on the commercial XOS preparations used in this study, suggesting that its native substrate may differ from the dominant substrates present in these preparations. One possibility is that XouC acts on XOS with alternative linkage structures, such as β-1,3-linkages found in side chains of corn glucuronoarabinoxylan or red algae.^28^ ^,^ ^62^ However, testing this possibility will require access to defined substrates that are not currently (commercially) available.

**Figure 6.**
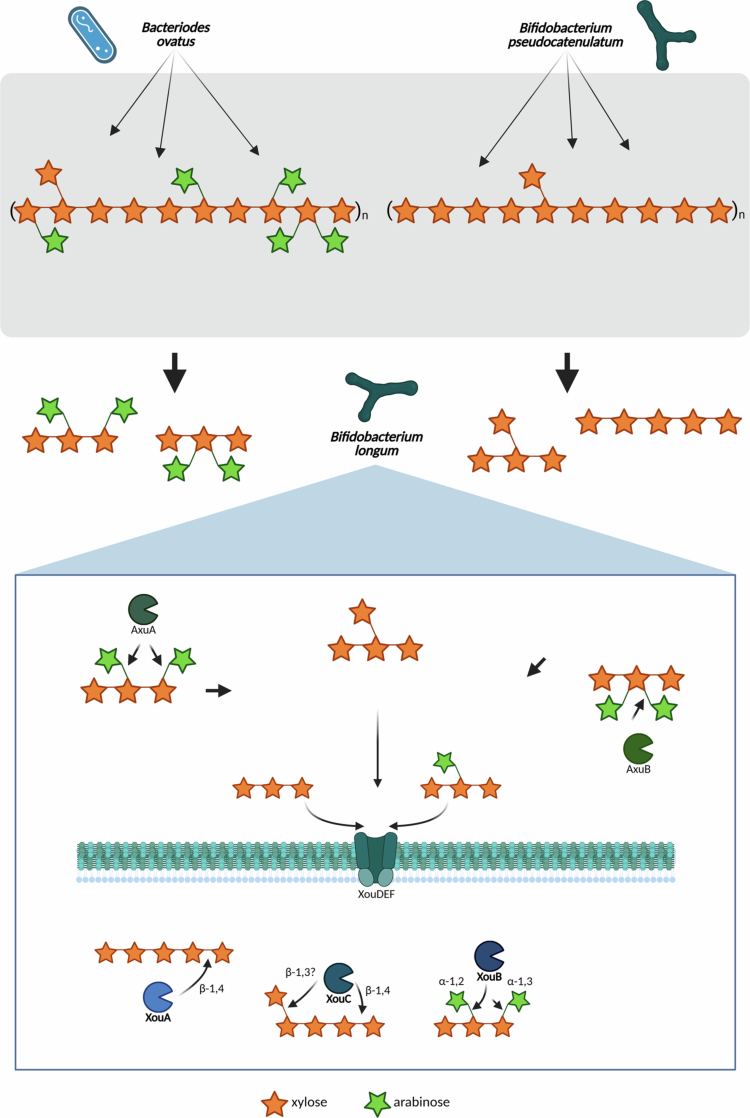
Model of XOS utilization by *B. longum* through the *xyl-xou* cluster. Complex xylan is first cleaved into XOS by a primary degrader, such as *Bacteriodes ovatus* or *Bifidobacterium pseudocatenulatum*, after which the released oligosaccharides can be utilized by *B. longum* strains carrying a functional *xyl-xou* cluster. We previously showed that two extracellular α-arabinofuranosidases cooperatively remove arabinose substitutions from rye- or wheat-derived arabinoxylan.^38^ Here, we show that XOS are imported into the cell by the XouDEF ABC-type uptake system, after which XouA, XouB, and XouC hydrolyze β-1,4 or α-1,2/α-1,3 linkages. Additional bonds and sugar moieties may be present in natural xylan-derived substrates that are not depicted in this model. Created in BioRender.

Neither XouA nor XouC hydrolyzed arabinose-substituted XOS, at least among the smaller branched substrates tested. XouB, by contrast, removed arabinose substitutions from singly substituted arabinoxylo-oligosaccharides (AXOS) containing α-1,2 or α-1,3 linkages, but did not hydrolyze doubly substituted AXOS. These activities suggest a model in which XouB removes arabinose substitutions from imported AXOS, enabling subsequent cleavage of the xylose backbone by XouA or XouC (Figure 6). *B. longum* NCIMB 8809 also encodes two extracellular α-arabinofuranosidases, AxuA and AxuB, that cleave α-1,2 or α-1,3 linkages from singly or doubly substituted substrates.^38^ Thus, extracellular debranching by AxuA/AxuB and intracellular debranching by XouB may act together to expand the range of xylan-derived oligosaccharides accessible to *B. longum*.

The requirement for *xouA* also extended to cross-feeding on xylan-derived substrates. Previous studies showed that *B. longum* can grow on xylan in the presence of primary degraders such as *B. pseudocatenulatum* or *B. ovatus.* ^28^ ^,^ ^30^ We confirmed xylan-mediated cross-feeding between *B. longum* NCIMB 8809 and *B. ovatus* CCUG 4943 and further showed that this interaction depends on *xouA*. Thus, although *B. longum* NCIMB 8809 cannot directly utilize xylan, it can benefit from xylan degradation by another species if it retains the intracellular machinery required to metabolize the released XOS. The structure of the released XOS likely depends on both the source of xylan and the enzymatic repertoire of the primary degrader, which may help explain why the *xyl-xou* cluster encodes multiple glycoside hydrolases with distinct substrate preferences.^28^ ^,^ ^30^ ^,^ ^38^

Our results also clarify the role of carbohydrate transport in XOS metabolism. The *xouDEF* genes encode a predicted ABC-type carbohydrate uptake system that is transcriptionally induced during growth on XOS and genetically required for XOS utilization. This transporter is distinct from the previously identified PenABCD system involved in xylose and pentose uptake,^56^ as disruption of *penD* did not impair growth on XOS. Conversely, disruption of *xouF* in *B. longum* NCIMB 8809 or a naturally occurring nonsense mutation in the *xouD* homolog of *B. longum* MM0289 abolished XOS utilization while leaving xylose metabolism intact. These findings support a model in which *B. longum* imports short-chain XOS through XouDEF and then degrades them intracellularly to xylose, which is subsequently metabolized through the XylA/XylB-dependent pathway into the bifid shunt.

Although xylose metabolism appears to be a conserved trait of *B. longum*, with all tested genomes encoding *xylA* and *xylB* ^56^, XOS utilization is not universal. Several strains lacked key components of the *xyl-xou* cluster, while others contained apparent homologs but carried transposon insertions or point mutations that likely disrupt function. This observation may explain why some *B. longum* strains, including NCC 2705,^31^ harbor genes predicted to encode XOS-active enzymes yet fail to grow on XOS. More broadly, these findings illustrate an important limitation of gene-trait matching based solely on gene presence or absence: mutations, mobile elements, and cluster integrity must also be considered. Experimental validation remains essential for assigning metabolic function from genome sequence alone.

Homologs of the *xyl-xou* gene cluster are present in several other bifidobacterial taxa, including *B. adolescentis*, *B. longum* subsp. *infantis, B. animalis* subsp. *lactis*, and *B. pseudocatenulatum.* ^63–65^ However, the presence of a homologous cluster does not necessarily imply that it is intact, expressed, or sufficient to support XOS utilization. In *B. longum*, the cluster is variably present and strains carrying the cluster do not form a single lineage in the core-genome phylogeny, suggesting repeated loss, disruption, or rearrangement. Such changes may reflect ecological contexts in which XOS utilization is no longer advantageous, for example because relevant dietary substrates are scarce or because primary degraders that release XOS are absent. Similar patterns have been observed for fucose and fucosyllactose utilization clusters, which are present in some but not all bifidobacterial species.^66–68^ The distribution of the *xyl-xou* cluster may therefore reflect horizonal gene transfer among bacteria sharing a gut niche, ancestral presence followed by lineage-specific loss, or a combination of both processes.

These findings have practical implications for the design of prebiotic and synbiotic strategies. XOS has been classified as a prebiotic because XOS-rich diets can increase the relative abundance of bifidobacteria.^8^ ^,^ ^69^ However, our results indicate that XOS utilization is strain specific, even within *B. longum*. Thus, pairing XOS with a *B. longum* strain should not assume that the strain can metabolize XOS; instead, the integrity and function of the *xyl-xou* cluster should be experimentally confirmed. This point is especially important for synbiotic development, where successful engraftment may depend on matching a strain’s glycan-utilization capacity to the dietary substrate provided.^70^ Future clinical studies using defined XOS preparations and genomically characterized *B. longum* strains will be needed to determine whether the *xyl-xou* cluster predicts strain expansion, persistence, and host-associated outcomes *in vivo*.

Overall, this study defines the genetic and enzymatic basis of XOS utilization in *B. longum* and shows that this capacity depends on an intact *xyl-xou* cluster encoding both an XOS uptake system and intracellular glycoside hydrolases. By linking strain-level genomic variation to carbohydrate utilization phenotypes, these findings provide a framework for understanding how dietary glycans shape bifidobacterial ecology and for designing more targeted prebiotic and synbiotic interventions.

## Supplementary Material

Supplementary Material XOS_Supplemental_information_revised clean.docx

## Data Availability

Sequencing data from this study are available through the NCBI BioProject database under accession PRJNA1082215, with associated Sequence Read Archive accessions SRR35316394, SRR35316395, and SRR35993047.
