# Supplementary material for: Genetic and enzymatic basis of xylooligosaccharide metabolism by Bifidobacterium longum
Source: Gut Microbes. 2026 Jul 10;18(1):2701440. doi: 10.1080/19490976.2026.2701440 (PMC13360511; doi:10.1080/19490976.2026.2701440)
Supplement: Supplementary Material — XOS_Supplemental_information_revised clean.docx [file KGMI_A_2701440_SM5245.docx]

**Supplemental information for “Genetic and enzymatic basis of xylo‐oligosaccharide metabolism by *Bifidobacterium longum*”**

**Authors:** Lisa Friess^1^, Fionnuala M. McAuliffe^2^, Paul D. Cotter^1,3^, Jose Munoz-Munoz^4^, Anthony L. Shiver^5^, Kerwyn Casey Huang^5,6,7^, Anne de Jong^8^, Douwe van Sinderen^1,*^

**Affiliations:**

^1^APC Microbiome Ireland & School of Microbiology, University College Cork, Western Road, Ireland

^2^UCD Perinatal Research Centre, School of Medicine, University College Dublin, National Maternity Hospital, Dublin, Ireland

^3^Teagasc Food Research Centre, Cork, Ireland

^4^Durham University, Durham, England, Great Britain

^5^Department of Bioengineering, Stanford University, Stanford, USA

^6^Department of Microbiology and Immunology, Stanford University School of Medicine, Stanford, USA

^7^Chan-Zuckerberg Biohub, San Francisco, USA

^8^Department of Molecular Genetics, University of Groningen, Groningen, the Netherlands

*Corresponding author: [d.vansinderen@ucc.ie](mailto:d.vansinderen@ucc.ie)

**Running title:** Xylooligosaccharide utilisation by *Bifidobacterium longum*

**Supplementary Tables**

**Table S1: Characterised enzymes with *in vitro* activity on XOS.**

| Strain | Enzyme | Enzyme description | GH family | Activity | Homologs | Amino acid similarity to NCIMB 8809 homologue | Reference |
| --- | --- | --- | --- | --- | --- | --- | --- |
| *B. adolescentis* LMG1050219 | RexA | Exo-oligo xylanase | GH8 | Hydrolysis of β-1,4-linked xylose residues from the reducing end of XOS | XylA | No significant similarity | ^1^ |
| *B. adolescentis* DSM20083 | XylA | Endo-1,4-β-xylanase | GH8 |  | RexA | No significant similarity | ^2^ |
| *B. adolescentis* ATCC15703 | XylB | β-xylosidase | GH120 | Hydrolysis of β-1,4-linked xylans to remove xylose residues from the non-reducing end of XOS | BP_  XUL2 | 46.74% over 99%(XouC) | ^3^ |
| *B. pseudocatenulatum*  YIT 4072 | BP_  XUL2 | β‑ xylosidase | GH120 |  | XylB | 49.62% over 100% (XouC) | ^4^ |
| *B. adolescentis* DSMZ 18350 |  | endo-1,4-β-xylanase | GH43 | Hydrolysis of β-1,4-xyloside bonds in XOS | BAD_  1527 | 25.44% over 31% (XouA) | ^5,6^ |
| *B. adolescentis* ATCC15703 | XylC | β-xylosidase | GH43 | Hydrolysis of β-1,4-linked xylose residues from the reducing end of XOS | BXA43 | 80.15% over 99% (XouA) | ^3^ |
| *B. animalis* subsp. *lactis* BB-12 | BXA43 | β-D-xylosidase/α-L-arabinofuranosidase | GH43 |  | XylC | 79.22% over 100% (XouA) | ^7^ |
| *B. breve*  K-110 |  | Exo-β-D-xylosidase | GH51 | Hydrolysis of β-1,4-linked xylose residues from the non-reducing end of XOS | N/A | No significant similarity | ^8^ |
| *B. adolescentis* | BaXyl5B | β-xylosidase | GH52 | Hydrolysis of β-1,4-linked xylose residues from the non-reducing end of XOS | N/A | N/A | ^9^ |

# Table S2: Bacterial strains and plasmids used in this study.

| **Strain/plasmid** | **Features** | **Reference/source** |
| --- | --- | --- |
| ***B. longum* subsp*. longum* strains** | | |
| NCIMB 8809 | isolated from infant stool | NCIMB/CP011964.1 |
| JCM 1217 | Isolated from adult stool | JCM/CP072501.1 |
| MB0044 | isolated from infant stool | CP146844^10^ |
| MB0212 | isolated from infant stool | CP157377–CP157378^10^ |
| MB0308 | isolated from infant stool | CP146700^10^ |
| MB0318 | isolated from infant stool | CP146699^10^ |
| MM0003 | isolated from adult stool | CP146904–CP146907^10^ |
| MM0024 | isolated from adult stool | CP146639–CP146641^10^ |
| MM0160 | isolated from adult stool | CP146698^10^ |
| MM0259 | isolated from adult stool | CP155475^10^ |
| MM0286 | isolated from adult stool | CP146642–CP146644^10^ |
| MM0289 | isolated from adult stool | CP146901–CP146903^10^ |
| MM0302 | isolated from adult stool | CP146696^10^ |
| MM0307 | isolated from adult stool | CP146695^10^ |
| MM0321 | isolated from adult stool | CP146636–CP146638^10^ |
| MM0360 | isolated from adult stool | CP146634–CP146635^10^ |
| MM0362 | isolated from adult stool | CP146630–CP146633^10^ |
| MM0364 | isolated from adult stool | CP146694^10^ |
| MM0369 | isolated from adult stool | CP146491–CP146492^10^ |
| MM0375 | isolated from adult stool | CP146647–CP146649^10^ |
| MM0380 | isolated from adult stool | CP146484^10^ |
| MM0441 | isolated from adult stool | CP146496–CP146497^10^ |
| MM0450 | isolated from adult stool | CP146493–CP146495^10^ |
| MM0464 | isolated from adult stool | CP146483^10^ |
| MM0465 | isolated from adult stool | CP146485^10^ |
| MM0492 | isolated from adult stool | CP146645–CP146646^10^ |
| MM0494 | isolated from adult stool | CP146487–CP146490^10^ |
| NCIMB 8809 + pBM5 | NCIMB 8809 containing plasmid pBM5 | This work |
| NCIMB 8809 ∆*xouA* + pBM5 | NCIMB 8809 with a disruption in *xouA* containing plasmid pBM5 | This work |
| NCIMB 8809 ∆*xouA* + pBM5::*xouA* | NCIMB 8809 with a disruption in *xouA* containing plasmid pBM5::*xouA* | This work |
| NCIMB 8809 ∆*xouB* + pBM5 | NCIMB 8809 with a disruption in *xouB* containing plasmid pBM5 | This work |
| NCIMB 8809 ∆*xouC* + pBM5 | NCIMB 8809 with a disruption in *xouC* containing plasmid pBM5 | This work |
| NCIMB 8809 Δ*xouF* + pBM5 | NCIMB 8809 with a disruption in *xouF* containing plasmid pBM5 | This work |
| NCIMB 8809 Δx*ouF* + pBM5::*xouF* | NCIMB 8809 with a disruption in *xouF* containing plasmid pBM5::*xouF* | This work |
| NCIMB 8809 Δx*ylA* + pBM5 | NCIMB 8809 with a disruption in *xylA* containing plasmid pBM5 | ^10^ |
| NCIMB 8809 Δx*ylA*+ pBM5::*xylA* | NCIMB 8809 with a disruption in *xylA* containing plasmid pBM5::*xylA* | ^10^ |
| NCIMB 8809 Δx*ylB* + pBM5 | NCIMB 8809 with a disruption in x*ylB* containing plasmid pBM5 | ^10^ |
| NCIMB 8809 Δx*ylB*+ pBM5::*xylB* | NCIMB 8809 with a disruption in *xylB* containing plasmid pBM5::*xylB* | ^10^ |
| NCIMB 8809 Δ*penD* + pBM5 | NCIMB 8809 with a disruption in *penD* containing plasmid pBM5 | ^10^ |
| MM0289 + pBM5 | MM0289 containing plasmid pBM5 | This work |
| MM0289 + pBM5::*xouD* | MM0289 containing plasmid pBM5::*xouD* | This work |
| **Other *Bifidobacterium* strains** | | |
| *B. longum* subsp. *infantis* CECT 7210 | isolated from infant stool | LN824140^11^ |
| *B.* *pseudocatenulatum* DSM 20438 | isolated from infant stool | DSMZ/AP012330 |
| *B. catenulatum* subsp. *kashiwanohense* DSM 21854 | isolated from infant stool | DSMZ/NZ_AP012327 |
| *B. adolescentis* DSM 20083 | isolated from adult stool | DSMZ/AP009256 |
| *B. animalis* subsp. *lactis* DSM 10140 | isolated from yogurt starter culture | DSMZ/CP001606 |
| *B. dentium* MB0114 | isolated from infant stool | CP162922^10^ |
| ***E. coli* strains** |  |  |
| EC101 | Cloning host, *repA*^+^ *Km^R^* | ^12^ |
| DH5α | Cloning host, *recA1* | Invitrogen |
| BL21 | Cloning host, T7^-^ | Stratagene |
| **Plasmids** |  |  |
| pET28b | *E.coli* expression vector with N-terminal His tag | Novagen |
| pET28b::*xouA* | pET28b harbouring *xouA* | This work |
| pET28b::*xouB* | pET28b harbouring *xouB* | This work |
| pET28b::*xouC* | pET28b harbouring *xouC* | This work |
| pFREM2 | pFREM28 derivative, R-M motif-free for *B. longum* | ^13^ |
| pFREM2::*xouA* | pFREM2 harbouring an internal fragment of *xouA* | This work |
| pFREM2::*xouB* | pFREM2 harbouring an internal fragment of *xouB* | This work |
| pFREM2::*xouC* | pFREM2 harbouring an internal fragment of *xouC* | This work |
| pFREM2::*xouF* | pFREM2 harbouring an internal fragment of *xouF* | This work |
| pBM5 | pBC1, pUC19-Tc^r^ | ^13^ |
| pBM5::*xouA* | pBM5 harbouring *xouA* and the p44 promoter | This work |
| pBM5::*xouD* | pBM5 harbouring *xouD* and the p44 promoter | This work |
| pBM5::*xouF* | pBM5 harbouring *xouF* and the p44 promoter | This work |
| pNZEM | Gene expression vector, Em^R^, harbouring EcoRII methylase from NCIMB 8809 | ^14^ |

**Table S3: Information used to clone genes into pET28b for protein purification.**

| **Gene** | **Locus tag** | **Length** | **Genome location** | **Primers** |
| --- | --- | --- | --- | --- |
| *xouA* | B8809_1434 | 1.6 kb | 1,798,016–1,799,625 | atgaagatctccaatcccgtac  gcctggcgtcatcatatttc |
| *xouB* | B8809_1433 | 1.8 bp | 1,796,151–1,797,932 | gtggcaagcgacaatgaac  caatattgtagagcgggctga |
| *xouC* | B8809_1431 | 2.2 kb | 1,793,409–1,795,389 | atgacgcaatatcatgtttccg  caacagtcgaaaccgcgatc |

**Table S4: Information used to construct gene disruption mutants.**

| **Gene** | **Locus tag** | **Insert length** | **Codons targeted** | **Genome location** | **Primers** |
| --- | --- | --- | --- | --- | --- |
| *xouA* | B8809_1434 | 392 bp | 12–143 of 552 | 1,799,220–1,799,612 | tgcggacccctcgatgattc  gctgcaccagatacttgcgtc |
| *xouB* | B8809_1433 | 493 bp | 34–199  of 590 | 1,797,357–1,797,850 | ctgggatgcgacgcgcgag  ggcgatgggctgcgtccag |
| *xouC* | B8809_1431 | 521 bp | 129–301 of 659 | 1,794,504–1,795,025 | gggcgatgtgtatctgaatgg  gcgccttgtagacggcc |
| *xouE* | B8809_1435 | 394 bp | 48–179 of 298 | 1,800,120–1,800,514 | ggcccggtgctgtac  ggatgatgatggcctgtg |

**Table S5:** **Information used for genetic complementation of gene disruption mutants.**

| **Gene** | **Locus tag** | **Artificial promoter?** | **Length** | **Genome location** | **Primers** |
| --- | --- | --- | --- | --- | --- |
| *xouA* | B8809_1434 | No | 3608 bp | 1,795,016–1,798,624 | cgcagcaaccataaccgtgc  gcctggcgtcatcatatttc |
| *xouD* | B8809_1437 | No | 1655 bp | 1,801,792–1,803,447 | cagagcgaatcagcccttg  ccgccttcatcagagaatgttg |
| *xouF* | B8809_1435 | Yes | 900 bp | 1,800,645–1,801,545 | ttcacaatgacgaccgc  gactagcccttgacagcac |

**Supplementary Figures**

**
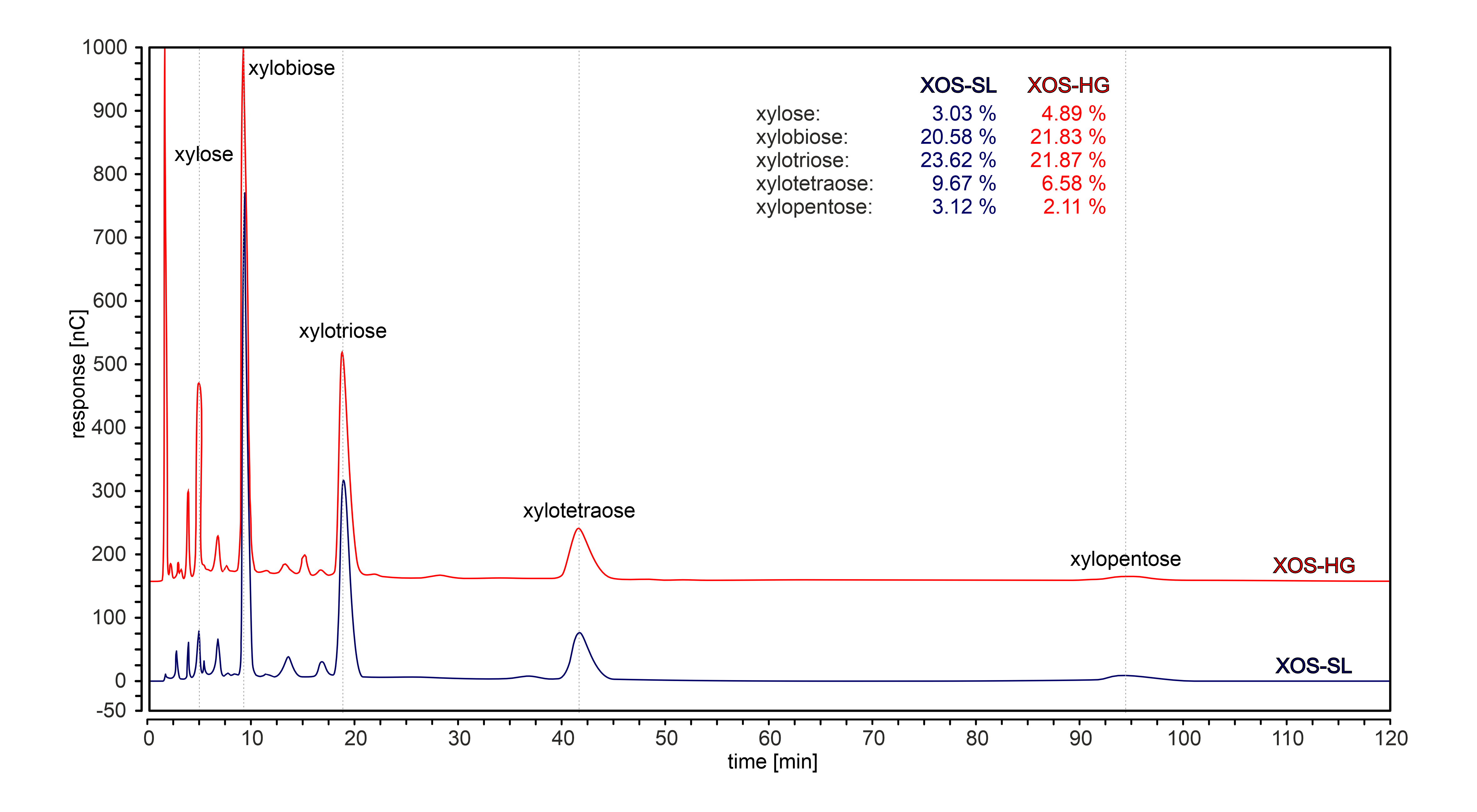
**

**Figure S1: Composition of commercial XOS preparations.**

**
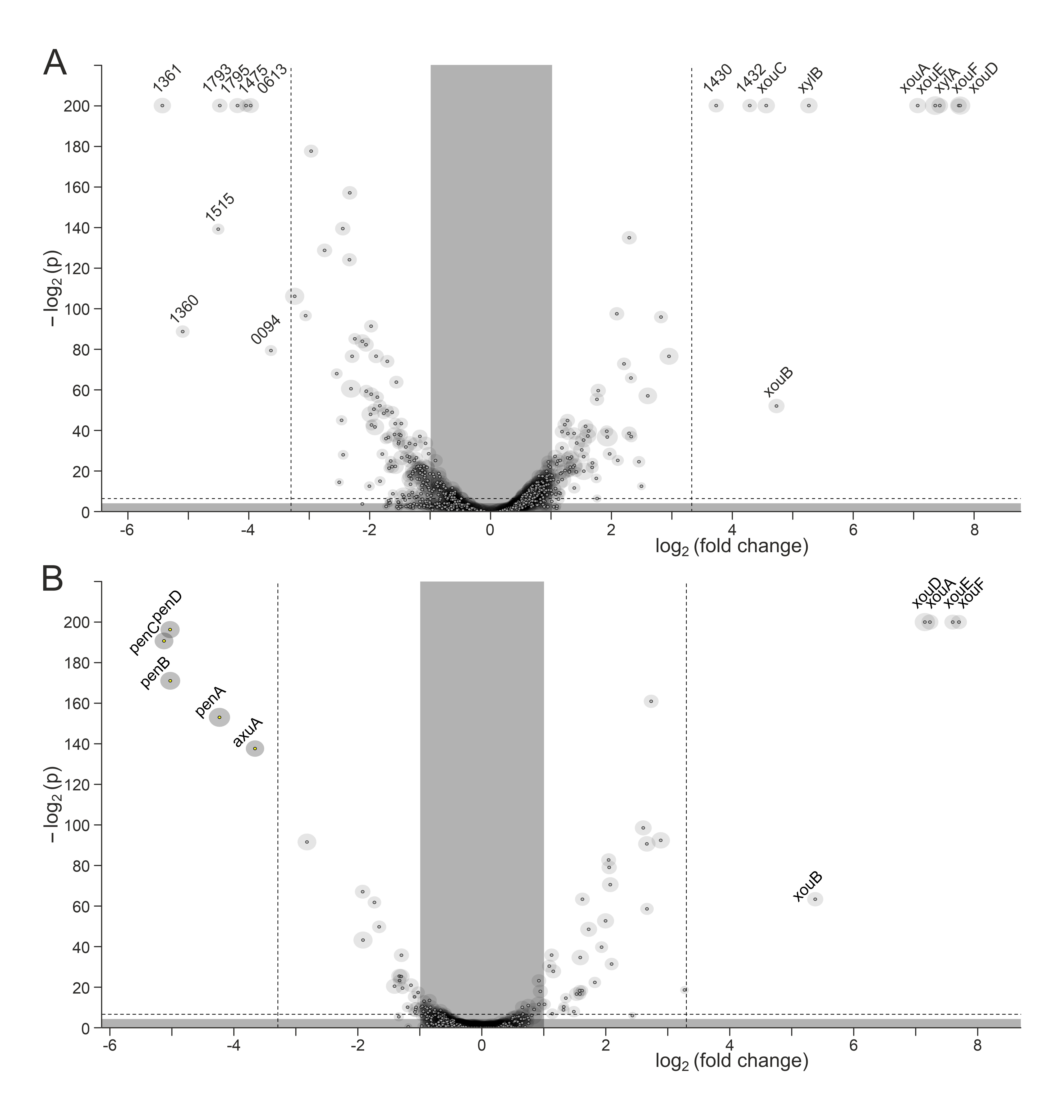
**

Figure S2: **Differential gene expression during growth of *B. longum* NCIMB 8809 on XOS.**

**
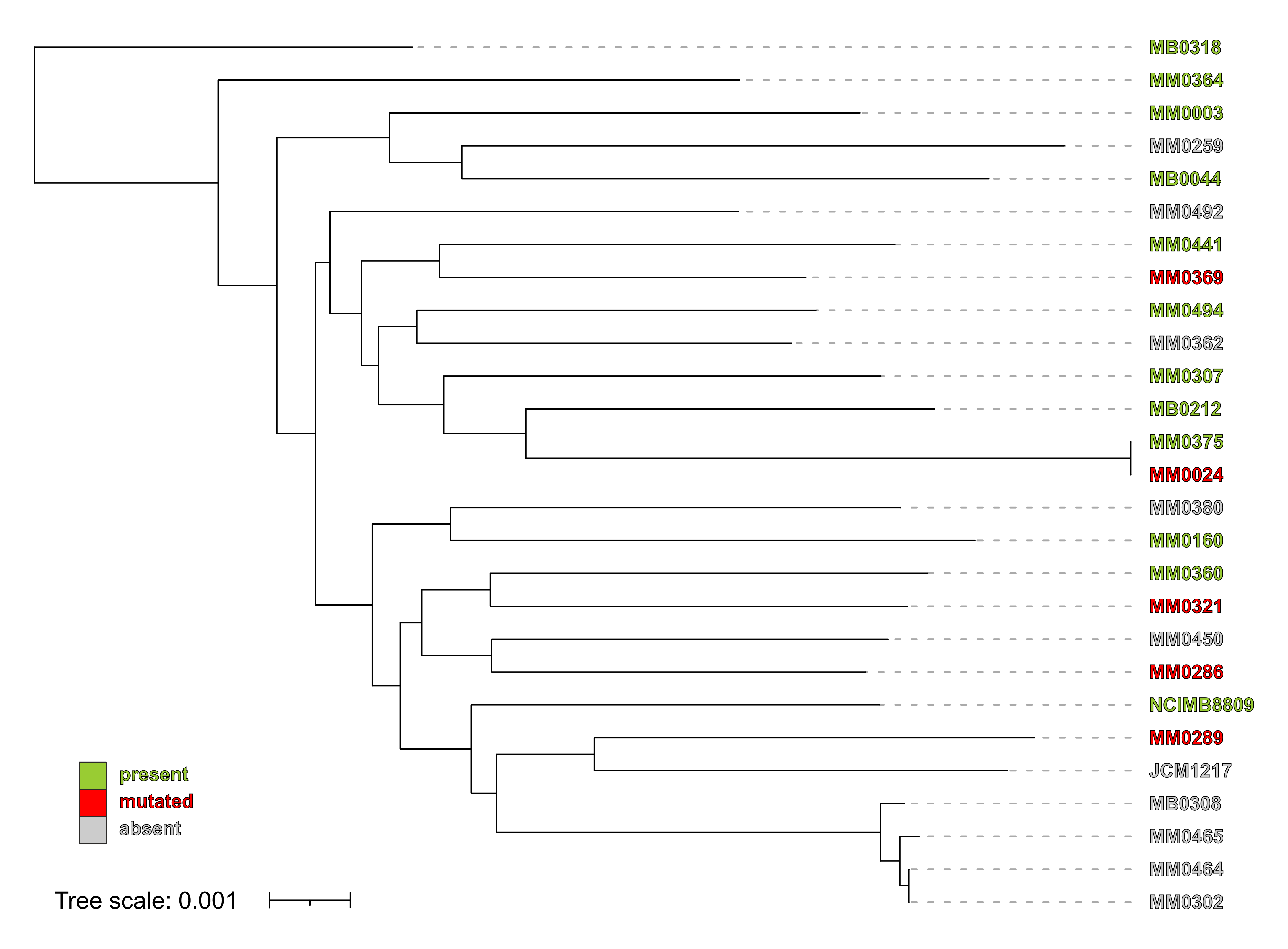
**

**Figure S3: Core-genome phylogeny 27 *B. longum* strains mapped by *xyl-xou* cluster status.**

**
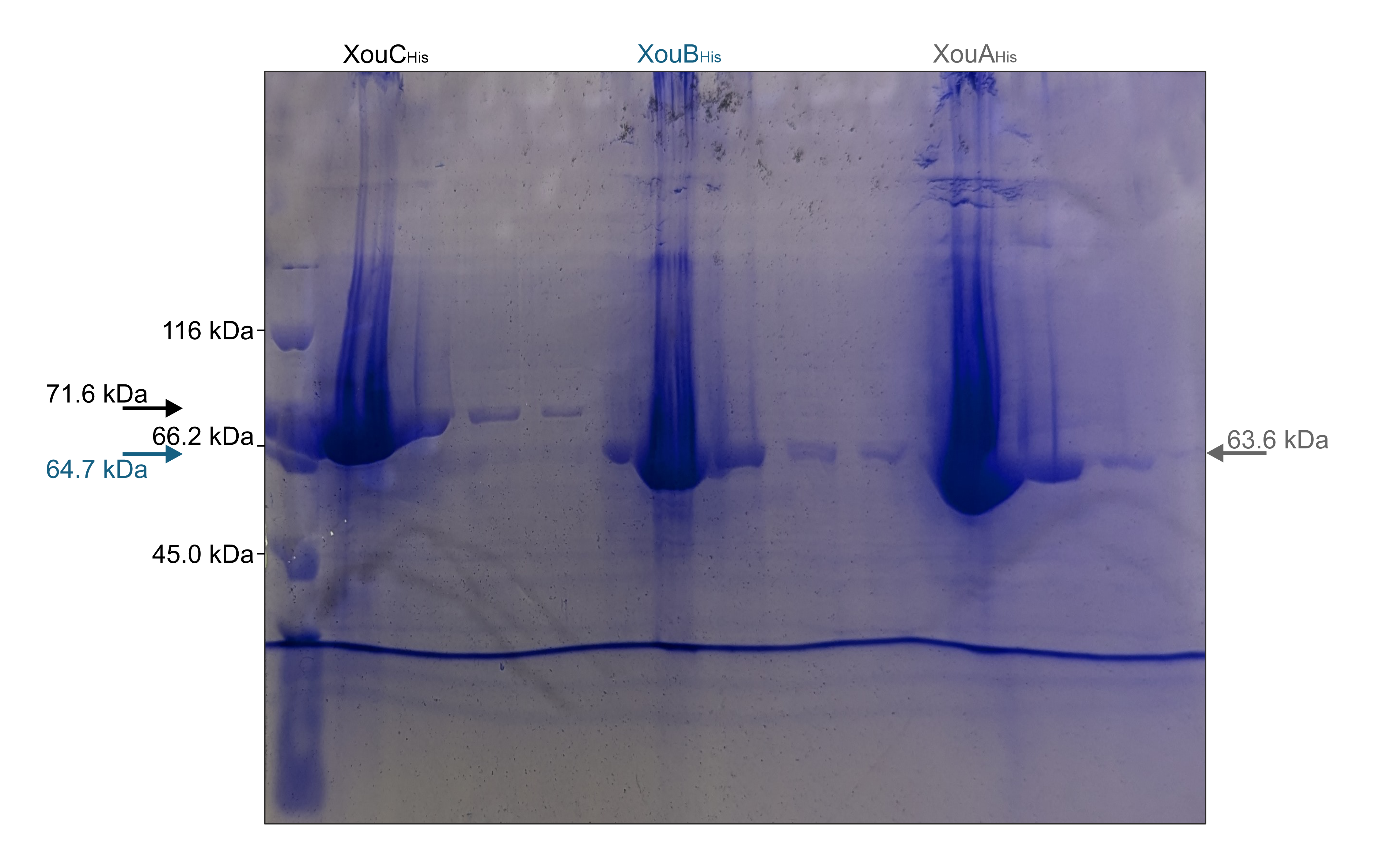
**

**Figure S4: SDS-PAGE analysis of purified XouA_His_, XouB_His_ and XouC_His_.**


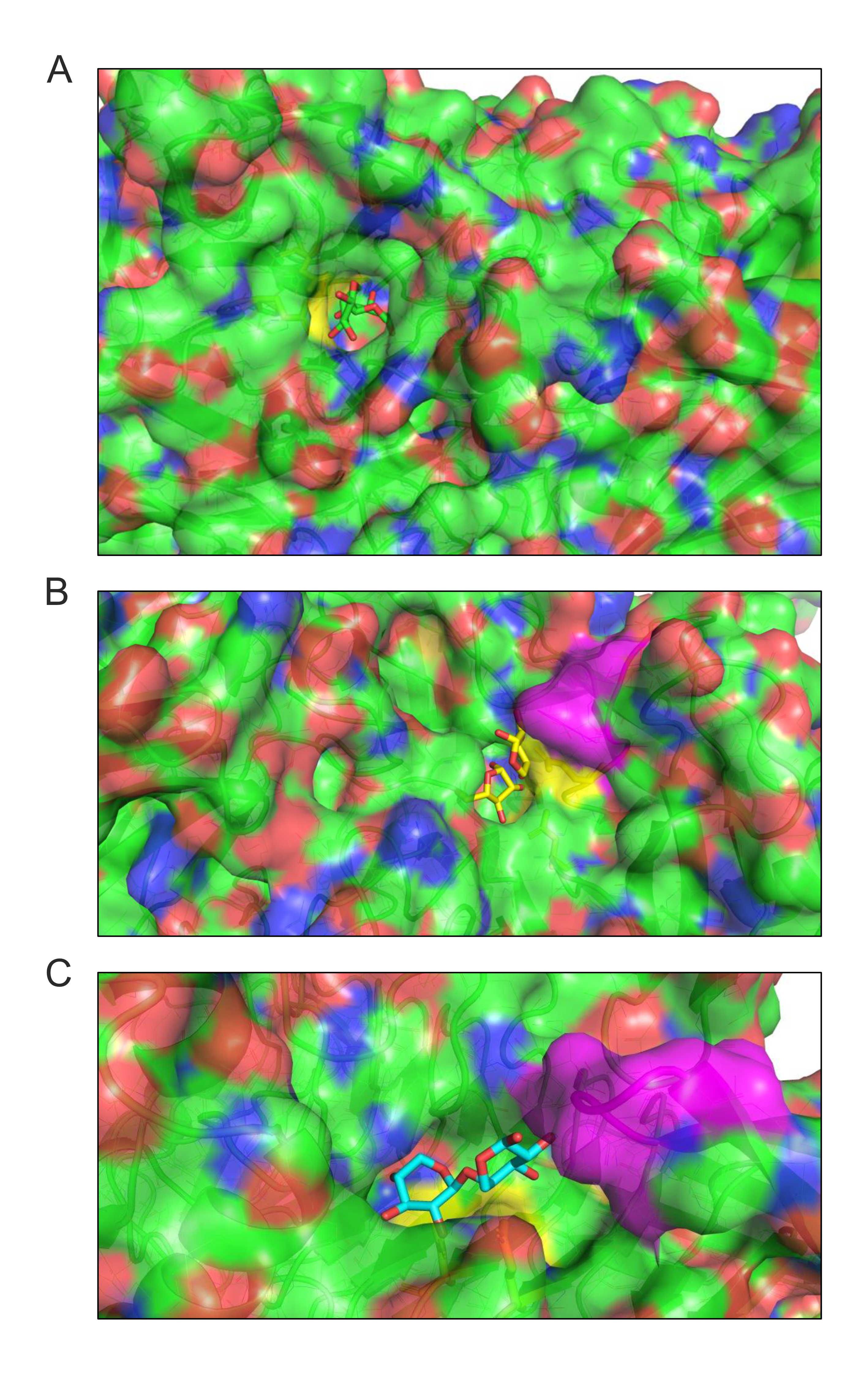


**Figure S5: AlphaFold3 models of XouA, XouB, and XouC substrate-binding sites.**


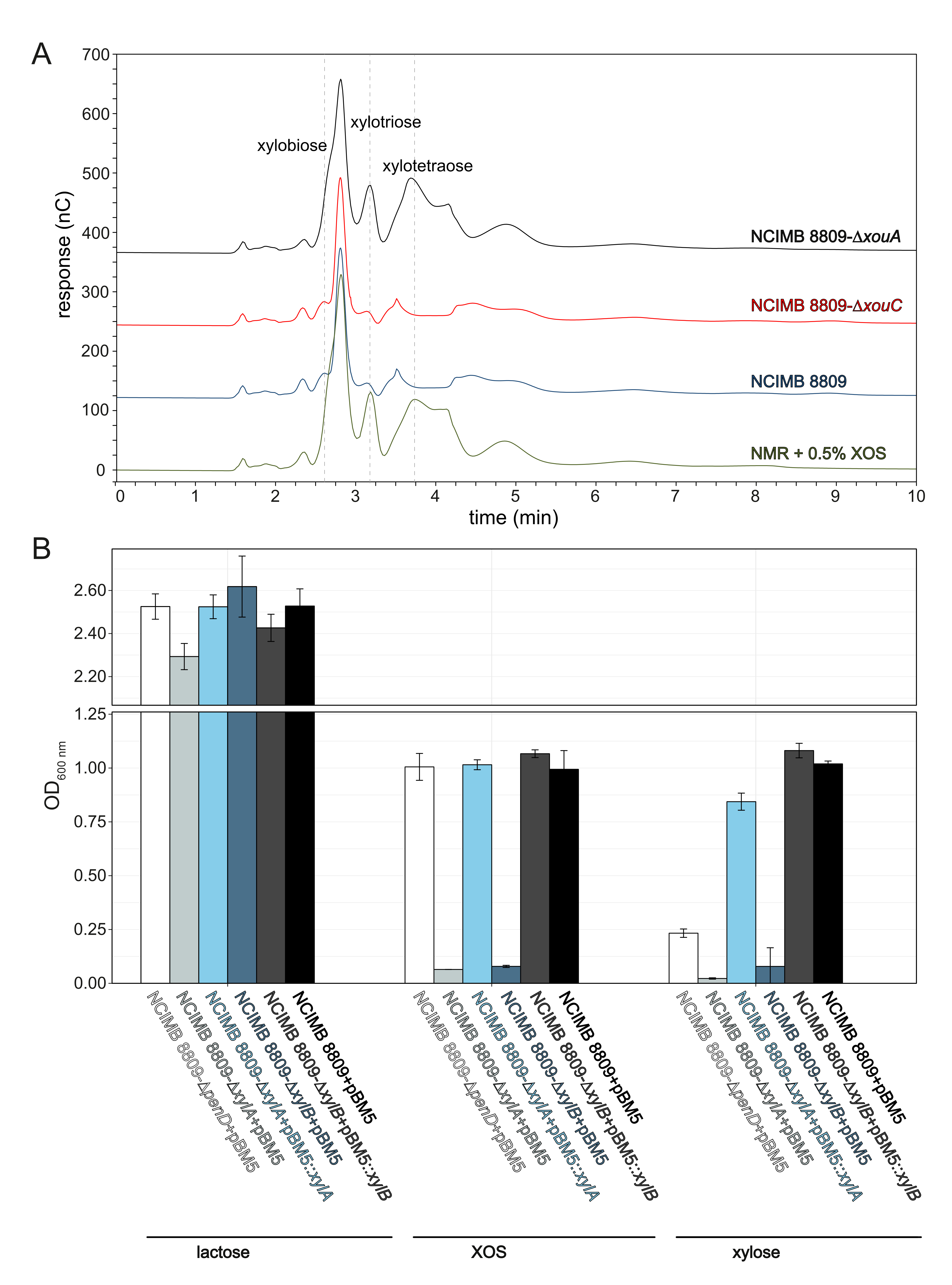


# Figure S6: XOS and xylose utilisation by *B. longum* NCIMB 8809 metabolic and transporter mutants.

**Supplementary Figure Legends**

**Figure S1: Composition of commercial XOS preparations.** Two commercial XOS preparations were analysed by HPAEC-PAD to determine their oligosaccharide composition: XOS-SL, provided by Shandong Longlive Bio-Technology Ltd. (blue), and XOS-HG, provided by van Wankum Ingredients and Henan Heagreen Bio-technology Co., Ltd. (red). Commercial standards were used to identify peaks corresponding to xylose, xylobiose, xylotetraose, and xylopentaose. The relative abundance of each component was calculated from the area under the corresponding peak using corresponding standards and is shown as the percentage of the total 10 mg XOS analysed.

Figure S2: **Differential gene expression during growth of *B. longum* NCIMB 8809 on XOS.** Volcano plots showing genes differentially expressed during growth on XOS compared with growth on lactose (A) or xylose (B). Labelled genes have an absolute log_2_(fold change) >3.5. Dashed lines indicate the thresholds used for labelling and significance.

**Figure S3: Core-genome phylogeny 27 *B. longum* strains mapped by *xyl-xou* cluster status.** Maximum-likelihood phylogeny based on the core-genome alignment of the 27 *B. longum* strains analysed in this study. Colours indicate *xyl-xou* cluster status: green, intact cluster present; grey, cluster absent; red, cluster present but disrupted by predicted loss-of-function mutations. The distribution of cluster status across the phylogeny suggests that *xyl-xou* cluster presence or disruption is not restricted to a single strain lineage**:** Colours map the presence or absent onto the tree. Green indicates presence, grey indicates absence, red indicates mutated cluster.

**Figure S4: SDS-PAGE analysis of purified XouA_His_, XouB_His_ and XouC_His_.** Purified XouA_His_, XouB_His_ and XouC_His_ proteins were analysed by sodium dodecyl sulphate-polyacrylamide gel electrophoresis (SDS-PAGE) using a 12.5% polyacrylamide gel and a Pierce™ Unstained Protein Molecular Weight Marker (14.4–116 kDa; Thermo Scientific, UK). The gel was run at 100 V for 2 h and stained with Coomassie Brilliant Blue. The observed molecular weights were consistent with the predicted molecular weights of 63.6 kDa for XouA_His_, 64.7 kDA for XouB_His_, and 71.6 kDa for XouC_His_.

**Figure S5: AlphaFold3 models of XouA, XouB, and XouC substrate-binding sites.** Structural models of the predicted binding sites of (A) XouC, (B) XouB and (C) XouA bound to their predicted ligands. Models were generated using AlphaFold3. Protein surfaces are shown with bound ligands displayed as sticks. Magenta regions highlight loops discussed in the text that may influence substrate accommodation and enzyme specificity.

# Figure S6: XOS and xylose utilisation by *B. longum* NCIMB 8809 metabolic and transporter mutants.

# (A) HPAEC-PAD analysis of cell-free supernatants after 24 h of growth in mMRS supplemented with 0.5% XOS. Compared with uninoculated medium containing XOS, wild-type NCIMB 8809 and NCIMB 8809-Δ*xouC* depleted the major XOS peaks, whereas NCIMB 8809-Δ*xouA* retained XOS components including xylobiose, xylotriose, and xylotetraose.

# (B) OD_600 nm_ after 24 h of anaerobic growth in mMRS supplemented with 0.5% (w/v) lactose, XOS, or xylose. Disruption of *xylA* or *xylB* reduced growth on both XOS and xylose, whereas disruption of *penD* reduced growth on xylose but not XOS.

# Supplementary References

1. Lagaert S, Van Campenhout S, Pollet A, Bourgois TM, Delcour JA, Courtin CM, Volckaert G. Recombinant expression and characterization of a reducing-end xylose-releasing exo-oligoxylanase from *Bifidobacterium adolescentis*. *Appl Environ Microbiol*. 2007;73(16):5374-5377. doi:10.1128/AEM.00722-07

2. Van Den Broek LAM, Lloyd RM, Beldman G, Verdoes JC, McCleary B V., Voragen AGJ. Cloning and characterization of arabinoxylan arabinofuranohydrolase-D3 (AXHd3) from *Bifidobacterium adolescentis*  DSM20083. *Appl Microbiol Biotechnol*. 2005;67(5):641-647. doi:10.1007/s00253-004-1850-9

3. Lagaert S, Pollet A, Delcour JA, Lavigne R, Courtin CM, Volckaert G. Characterization of two β-xylosidases from *Bifidobacterium adolescentis*  and their contribution to the hydrolysis of prebiotic xylooligosaccharides. *Appl Microbiol Biotechnol*. 2011;92(6):1179-1185. doi:10.1007/s00253-011-3396-y

4. Saito Y, Shigehisa A, Watanabe Y, Tsukuda N, Moriyama-Ohara K, Hara T, Matsumoto S, Tsuji H, Matsuki T, Saito CY, et al. Multiple Transporters and Glycoside Hydrolases Are Involved in Arabinoxylan-Derived Oligosaccharide Utilization in *Bifidobacterium pseudocatenulatum*. Published online 2025. doi:10.1128/AEM

5. Amaretti A, Bernardi T, Leonardi A, Raimondi S, Zanoni S, Rossi M. Fermentation of xylo-oligosaccharides by Bifidobacterium adolescentis DSMZ 18350: Kinetics, metabolism, and β-xylosidase activities. *Appl Microbiol Biotechnol*. 2013;97(7):3109-3117. doi:10.1007/s00253-012-4509-y

6. Fathallah W, Puchart V. The Bifidobacterium adolescentis BAD_1527 gene encodes GH43_22 α-L-arabinofuranosidase of AXH-m type. *AMB Express*. 2024;14(1):83. doi:10.1186/s13568-024-01738-9

7. Viborg AH, Sørensen KI, Gilad O, Steen-Jensen DB, Dilokpimol A, Jacobsen S, Svensson B. Biochemical and kinetic characterisation of a novel xylooligosaccharide-upregulated GH43 β-D-xylosidase/α-L-arabinofuranosidase (BXA43) from the probiotic *Bifidobacterium animalis* subsp. *lactis* BB-12. *AMB Express*. 2013;3:1-8. doi:10.1186/2191-0855-3-56

8. Shin HY, Lee JH, Lee JY, Han YO, Joo HAN M, Kim DH. *Isolation of Ginsenoside Ra1-Hydrolyzing Bacteria from Human Intestinal Microflora Bacterial Strains Pre-Viously Isolated from the Fresh Feces of a Healthy Korean Man Purification and Characterization of Ginsenoside Ra-Hydrolyzing b b-D-Xylosidase from* Bifidobacterium Breve*K-110, a Human Intestinal Anaerobic Bacterium Chart 1. Proposed Metabolic Pathway of Ginsenosides Ra1 and Ra2 by b-D-Xylosidase from* B. Breve *K-110*. Vol 26.; 2003.

9. Zhao J, Zhang B, Shen Y, Zhou J, Li Y, Hu Y. Cloning and characterization of a novel recombinant multifunctional glycoside hydrolase β-D-xylosidase/α-L-arabinopyranosidase/β-glucosidase from *Bifidobacterium adolescentis* and its application on the biotransformation of ginsenoside Rb3. *Biocatal Biotransformation*. 2025;43(1):97-108. doi:10.1080/10242422.2024.2421005

10. Friess L, McAuliffe FM, Cotter PD, Shiver AL, Huang KC, de Jong A, van Sinderen D. Metabolic pathway analysis reveals hierarchical pentose sugar utilization and metabolic flexibility of *Bifidobacterium longum*. *Gut Microbes*. 2026;18(1):2647591. doi:https://doi.org/10.1080/19490976.2026.2647591
